# CBL1/CIPK23 phosphorylates tonoplast sugar transporter TST2 to enhance sugar accumulation in sweet orange (*Citrus sinensis*)

**DOI:** 10.1111/jipb.13812

**Published:** 2024-11-29

**Authors:** Mengdi Li, Zuolin Mao, Zeqi Zhao, Siyang Gao, Yanrou Luo, Ziyan Liu, Xiawei Sheng, Xiawan Zhai, Ji‐Hong Liu, Chunlong Li

**Affiliations:** ^1^ National Key Laboratory for Germplasm Innovation & Utilization of Horticultural Crops, College of Horticulture and Forestry Sciences Huazhong Agricultural University Wuhan 430070 China; ^2^ Hubei Hongshan Laboratory Wuhan 430070 China

**Keywords:** citrus, CsCBL1/CIPK23, phosphorylation, tonoplast sugar transporter, vacuole

## Abstract

Fruit taste quality is greatly influenced by the content of soluble sugars, which are predominantly stored in the vacuolar lumen. However, the accumulation and regulation mechanisms of sugars in most fruits remain unclear. Recently, we established the citrus fruit vacuole proteome and discovered the major transporters localized in the vacuole membrane. Here, we demonstrated that the expression of *tonoplast sugar transporter 2* (*CsTST2*) is closely associated with sugar accumulation during sweet orange (*Citrus sinensis*) ripening. It was further demonstrated that CsTST2 had the function of transporting hexose and sucrose into the vacuole. Overexpression of *CsTST2* resulted in an elevation of sugar content in citrus juice sac, calli, and tomato fruit, whereas the downregulation of its expression led to the reduction in sugar levels. CsTST2 was identified as interacting with CsCIPK23, which binds to the upstream calcium signal sensor protein CsCBL1. The phosphorylation of the three serine residues (Ser277, Ser337, and Ser354) in the loop region of CsTST2 by CsCIPK23 is crucial for maintaining the sugar transport activity of CsTST2. Additionally, the expression of *CsCIPK23* is positively correlated with sugar content. Genetic evidence further confirmed that calcium and CsCIPK23‐mediated increase in sugar accumulation depends on CsTST2 and its phosphorylation level. These findings not only unveil the functional mechanism of CsTST2 in sugar accumulation, but also explore a vital calcium signal regulation module of CsCBL1/CIPK23 for citrus sweetness quality.

## Graphical abstract

The tonoplast‐localized sugar transporter CsTST2 has sucrose and hexose transporter activity and contributes to sugar accumulation in the vacuole. Furthermore, the calcium sensor complex CsCBL1/CIPK23 interacts with and phosphorylates CsTST2 to regulate its transporter activity, which in turn controls the sugar content in citrus fruit.

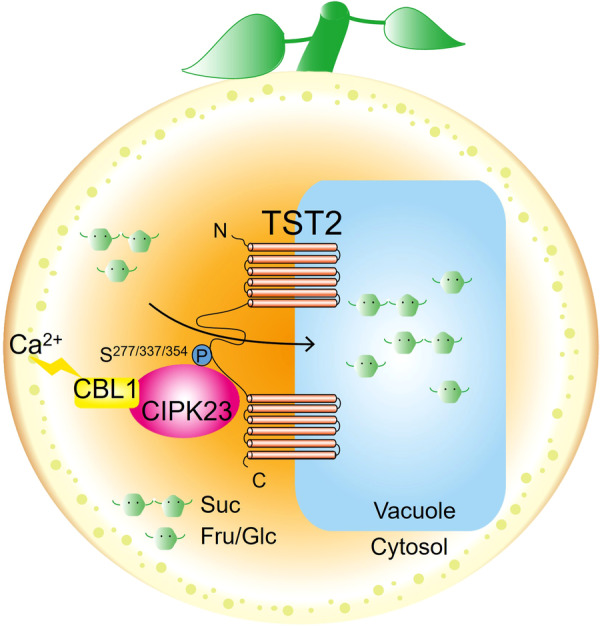

## INTRODUCTION

Sweet orange (*C. sinensis*) is a major cultivated species of *Citrus*, which holds the highest production among all fruit crops over the world. Due to the wide range of flavor and high nutritional content, sweet orange contributed to around 60% of the overall citrus output, encompassing both fresh fruit and orange juice consumption (Xu et al., 2013). The taste quality plays a pivotal role in shaping consumer preferences. The composition and content of soluble sugars are key determinants influencing fruit sweetness (Goldenberg et al., 2017; Chen et al., 2021). Generally, the soluble sugars found in citrus fruits typically consist of two types of hexoses (glucose, fructose) and sucrose (Feng et al., 2021). It should be pointed out that the vacuole is the primary site for the accumulation of soluble sugars, which contributes to the final sugar content and the sweetness quality of ripe fruit (Martinoia, 2018; Jiang et al., 2021; Liu et al., 2023). However, the knowledge of the process and mechanisms underlying the vacuolar sugar accumulation in citrus remain limited.

The large central vacuole is one of the important organelles that distinguish plant cells from animal cells (Shimada et al., 2018). Reports indicate that vacuoles are employed as an “intermediate storage compartment” for ions, small molecules, metabolites and a regular exchange/transport of those compounds between the cytosol and vacuolar lumen (Shitan and Yazaki, 2020). For flesh fruit, the large central vacuole contains most of the carbohydrates, organic acids and secondary metabolites that influence the nutritional value and flavor quality of the fruit (Liu et al., 2023). However, the majority of compounds present in vacuoles are hydrophilic and cannot freely diffuse across the vacuolar membrane. Consequently, tonoplast‐localized transporters or channels are required to facilitate material exchange through the vacuolar membrane (Martinoia et al., 2007; Martinoia, 2018). For instance, the tonoplast dicarboxylic transporters (tDTs) and vacuolar aluminum‐activated malate transporters (ALMTs) have been reported to exhibit malic acid transport activity, thereby contributing to the accumulation of acids in apple (Li et al., 2020b, 2024), grape (De Angeli et al., 2013), tomato (Sasaki et al., 2016; Ye et al., 2017), and other fruit crops. The soluble sugar content in sugar beet (Jung et al., 2015), apple (Zhu et al., 2020, 2023), watermelon (Ren et al., 2018), and peach fruit (Peng et al., 2020) was reported to be dependent on the expression of the tonoplast‐localized sugar transporters.

Considering the vast array of metabolic components, the most functional transporters or regulation mechanisms involved in the accumulation of metabolites in the fruit vacuole remain unidentified. With the continuous technological advancement, it has become feasible to investigate plant vacuolar transporters through the implementation of intact vacuole isolation and vacuolar proteomic analysis in fruit crops, such as in grape and fig flesh tissues (Kuang et al., 2019, 2022). Recently, we developed an effective methodology for isolating vacuoles from citrus fruits and obtained a comprehensive proteomic of citrus fruit vacuoles. The correlation analysis between sugar concentration and proteomic data identified the candidate tonoplast sugar transporters (TSTs) responsible for the accumulation of soluble sugar in citrus (Mao et al., 2024). However, the physiological function, transport activity, selective substrates and molecular mechanisms of the TST proteins require further investigation to unveil the process of sugar accumulation in citrus fruits.

Increasing studies have reported on the impact of agri‐environmental factors, such as mineral nutrition, water supply, or temperature, on sugar accumulation in citrus fruit. Among these, calcium ion (Ca^2+^) not only plays a crucial role as an essential mineral nutrient in plant development, but also serves as a universal signal in numerous physiological processes (Tang et al., 2020; Tian et al., 2020). In actuality, the decoding of Ca^2+^ signaling is complicated and requires sensor proteins. The calcium binding sensors with the conserved EF‐hand motifs have been extensively reported, including calmodulin (CaM), CaM‐like proteins (CMLs), Ca^2+^‐dependent protein kinases (CDPKs or CPKs), and calcineurin B‐like proteins (CBLs) coupled with their interacting kinases (CIPKs) (Tian et al., 2020; Luan and Wang, 2021). It is noteworthy that previous studies have demonstrated the impact of calcium sensors on ions or metabolites transport by binding and phosphorylating specific membrane transporters in various species. For instance, phosphorylation of iron‐regulated transporter IRT1 at the Ser149 residue by AtCPK21/23 increased the uptake of Fe under Fe‐deficient conditions (Wang et al., 2023). The manganese (Mn) vacuolar transporter MTP8 was undergoes an activation/deactivation fine‐tuning mechanism for manganese homeostasis via Ca^2+^‐dependent successive phosphorylation (Ju et al., 2022).

In addition, the calcium signaling and its sensor proteins were reported to be involved in the stress response and sugar homeostasis via regulation of the sugar transporters in horticultural plants. For example, CIPK13 and CIPK22 were reported to phosphorylate the sugar transporter MdSUT2.2, which encouraged sugar content and improved salt and drought resistance in apple plants (Ma et al., 2018a, 2018b). TST2 contributed to sugar homeostasis and drought tolerance under phosphorylation by the CPK27 in tomato plants (Zhu et al., 2024). The natural variations in the promoter region of *PbCPK28* resulted in the alteration of fructose levels in pear fruit through modulation of *PbCPK28* expression and phosphorylation of vacuolar sugar transport PbTST4 and hydrogen proton pump PbVHA‐A1 (Li et al., 2023a). Moreover, calcium concentration was reported to be increased during citrus ripening, which has a positive correlation with the soluble sugar content (Zhou et al., 2018). Hence, a relevant question is whether calcium signaling is involved in vacuolar sugar accumulation for citrus fruit sweetness quality, and what molecular mechanism underlies this process?

In this work, we proved that CsTST2 has both sucrose and hexose transport activity and contributes to the sugar accumulation in citrus fruit. Furthermore, the calcium sensor complex CsCBL1/CIPK23 was discovered to interact with and phosphorylate CsTST2 to regulate its transport activity, which in turn controlled sugar content. These findings provide insight into the molecular basis for calcium‐mediated flavor quality formation of citrus fruit.

## RESULTS

### The expression of TST *CsTST2* is associated with soluble sugar content during the citrus fruit development period

**Figure 1:**
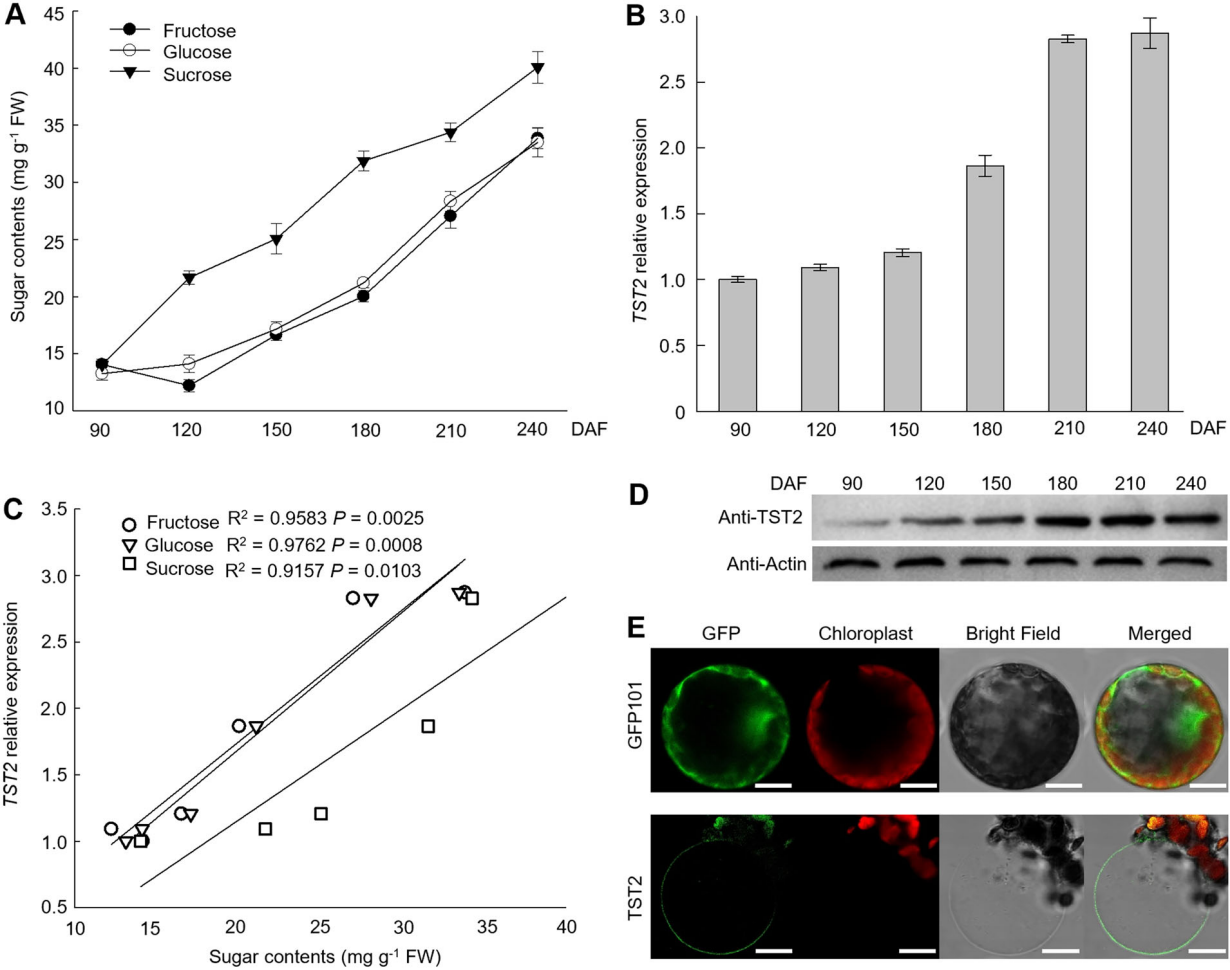
The tonoplast‐localized sugar transporter 2 (TST2) is associated with the sugar accumulation in citrus fruit **(A)** The sugar content during citrus fruit development. Values represent the mean ± *SE* with three biological replicates. **(B)** The transcript level of *CsTST2* during the citrus fruit development. Values represent the mean ± *SE* with three biological replicates. **(C)** Correlation analysis of *CsTST2* expression and sugar accumulation based on contents of fructose, glucose, sucrose and expression values of *CsTST2* at six developing stages of citrus fruits. Each developmental stage was assessed with three biological replicates. The Pearson correlation coefficient was applied for the correlation analysis between *TST2* genes and three sugars’ contents. *P* < 0.05 represents a significant correlation (two‐sided). **(D)** The protein level of CsTST2 was detected by its specific antibody during citrus fruit development. β‐actin was used to monitor equal sample loading. **(E)** The CsTST2 protein was localized in the vacuole membrane. Images of confocal microscopy were mesophyll protoplast and an isolated vacuole of tobacco expressing the empty vector control and CsTST2 tagged with green fluorescent protein, respectively. Scale bars = 10 μm. FW in **(A, C)**: fresh weight; DAF in **(A, B, D)**: day after flowering.

Based on recent vacuolar proteomic data, we identified the candidate TST in citrus fruit (Mao et al., 2024). Two TST paralogs were discovered from the reference genome of sweet orange (*Citrus sinensis*) in Citrus Pan‐genome to Breeding Database (CPBD; http://citrus.hzau.edu.cn/). And these two members are defined as CsTST1 and CsTST2 according to the phylogenetic analysis within the reported TST members from *Arabidopsis thaliana*, apple (*Malus domestica*), grape (*Vitis vinifera*), melon (*Cucumis melo*), watermelon (*Citrullus lanatus*) and sugar beet (*Beta vulgaris*) (Figure S1A). To determine the putative relationship between *CsTSTs* expression and sugar levels, we evaluated the sugar content and the transcriptional patterns of *CsTST1* and *CsTST2* in the different developing stages of citrus fruit. The results demonstrated that the amounts of fructose, glucose, and sucrose were gradually increased, which were positively correlated with the transcript level of *CsTST2*, but not *CsTST1* during fruit development (Figures 1A–C, S1B). The protein levels of CsTST2 were also increased with fruit ripening (Figure 1D). We further confirmed the precise vacuolar membrane localization of CsTST2 by observing intact green‐fluorescing vacuoles released from plant protoplasts expressing the CsTST2‐GFP (green fluorescent protein) fusion protein (Figure 1E). The results implied the potential function of CsTST2 for soluble sugar accumulation in citrus fruit vacuoles.

### CsTST2 has hexose and sucrose transport activity

**Figure 2:**
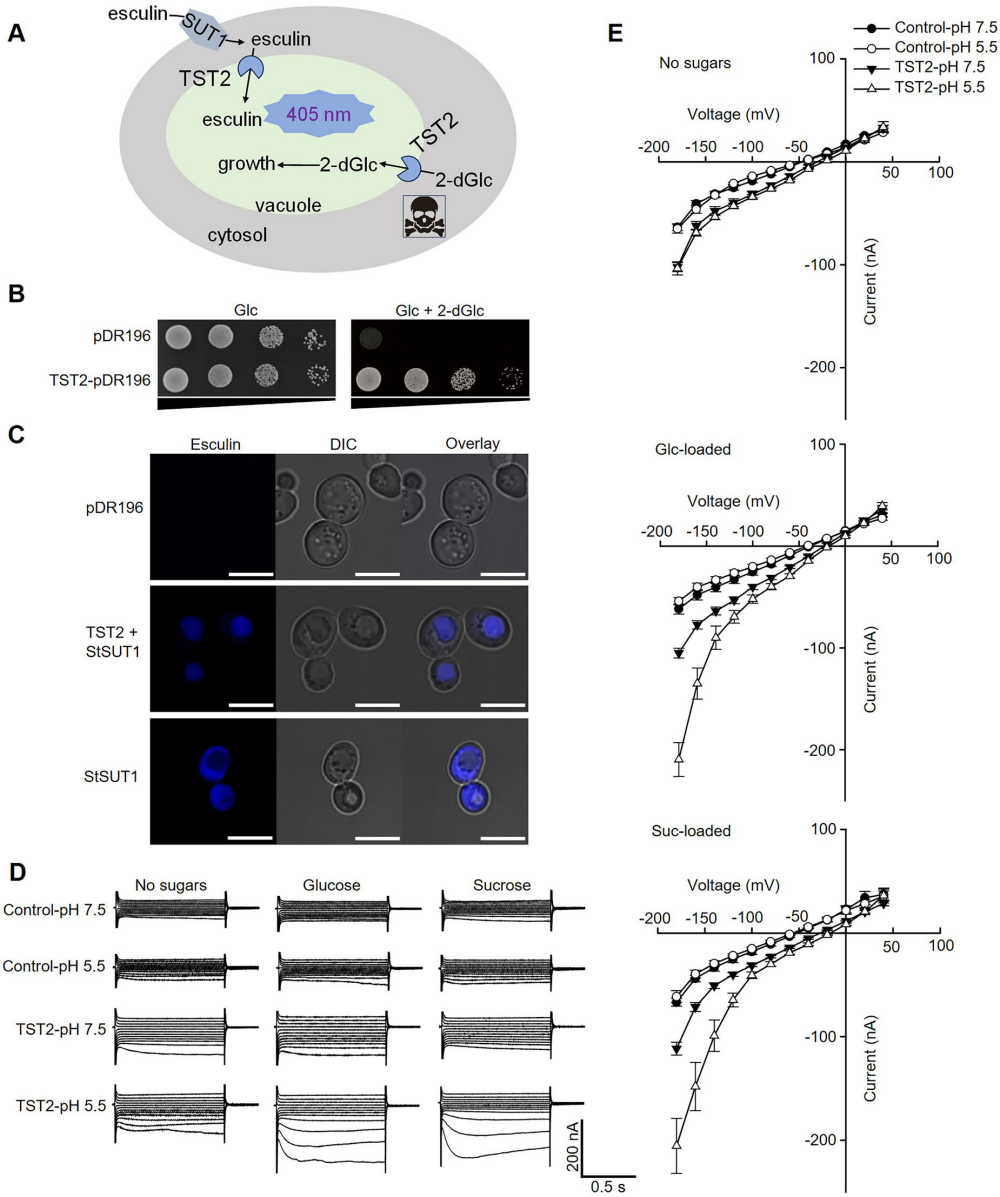
***Citrus sinensis*** **tonoplast‐localized sugar transporter 2 (CsTST2) has hexose and sucrose transport activity** **(A)** The esculin and 2‐dGlc uptake model in yeast cells. **(B)** The 2‐dGlc transport and yeast growth assay. The droplet test was conducted using yeast W303 cells harboring either the empty control vector (pDR196) or the construct vector expressing *CsTST2*. The accumulation of 2‐dGlc in the cytoplasm is known to be toxic and inhibits yeast growth. However, when *CsTST2* is expressed, yeast cells are able to transport 2‐dGlc into the vacuole, leading to recovery of yeast growth even in a medium containing 2‐dGlc. **(C)** The esculin uptake assay in yeast W303 cells. The esculin signal was labeled as cyan under a confocal microscope. Esculin was internalized into the cytosol upon expression of *StSUT1*, which encodes a sucrose transporter localized in the plasma membrane. The cyan fluorescence signal was predominantly concentrated within vacuoles when cells co‐expressed *StSUT1* and *CsTST2*. No signal was detected in the empty control cells. Scale bars = 5 μm. **(D)** Examples of voltage‐clamp recording currents elicited by control, *CsTST2*‐expressing cells with sugar loaded or not in *Xenopus laevis* oocytes. Experimental conditions, with intracellular glucose or sucrose and pH 5.5 or pH 7.5 in the bath buffer. **(E)** The current–voltage (*I*/*V*) relationships constructed from current recordings as those shown in **(D)**, for control‐pH 7.5 cells no sugar (*n* = 13), control‐pH 7.5 cells with glucose (*n* = 10) and control‐pH 7.5 cells with sucrose (*n* = 10); control‐pH 5.5 cells no sugar (*n* = 12), control‐pH 5.5 cells with glucose (*n* = 11), and control‐pH 5.5 cells with sucrose (*n* = 11); cells expressing *CsTST2‐*pH 7.5 no sugar (*n* = 13), *CsTST2‐*pH 7.5 with glucose (*n* = 10), and *CsTST2‐*pH 7.5 with sucrose (*n* = 15); cells expressing *CsTST2‐*pH 5.5 no sugar (*n* = 11), *CsTST2‐*pH 5.5 with glucose (*n* = 11), and *CsTST2‐*pH 5.5 with sucrose (*n* = 10). All of data are mean ± *SE* with indicated repeat number of cells.

To characterize the transport properties of CsTST2, the sugar uptake assay in yeast system was applied. Frist, the sub‐cell vacuolar localization of CsTST2 in yeast cells was detected via the GFP signals (Figure S2). According to a previous report (Patzke et al., 2019), 2‐deoxyglucose (2‐dGlc) and esculin were separately used to simulate hexose and sucrose transport in yeast strain system (Figure 2A). As shown in Figure 2B, the yeast cells with the transformation of *CsTST2* can grow well in 2‐dGlc medium, which is typically cytotoxic, undergoing detoxification through its sequestration within the vacuole (Weiss and Hiltbrand, 1985). Moreover, the esculin fluorescence signal was observed in the yeast cytosol region upon expression of a sucrose transporter *StSUT1*. However, when co‐expressed with *StSUT1* and *CsTST2*, the esculin signal translocated to the vacuole (Figure 2C). The results in yeast cells demonstrated that CsTST2 was able to transport monosaccharides and sucrose into vacuoles. The function of CsTST2 was further investigated by heterologously expressing it in *Xenopus laevis* oocyte cells and examining its electrophysiological properties. Analysis of the complementary RNA (cRNA)‐injected oocyte cells by fluorescent microscopy revealed that the CsTST2‐YFP (yellow fluorescent protein) fluorescence co‐localized with the plasma membrane deep red signal (Figure S3). Using the two‐electrode voltage‐clamp (TEVC) technique, the current was not changing in control cells upon loading of sugars under both pH 5.5 and pH 7.5 conditions, whereas oocytes expressing CsTST2 (no YFP tag) presented an increased current loaded with glucose or sucrose under pH 5.5 bath solution, but not at pH 7.5 (Figure 2D, E). In parallel, the transport assay of ^13^C‐labeled sucrose and glucose using the oocyte system demonstrated that cells expressing CsTST2 effluxed more sugars compared to control cells under pH 5.5 conditions (Figure S4). The findings demonstrated that CsTST2 was likely operating as an H^+^/sugars antiporter.

### Elevated levels of CsTST2 enhance sugar accumulation

**Figure 3:**
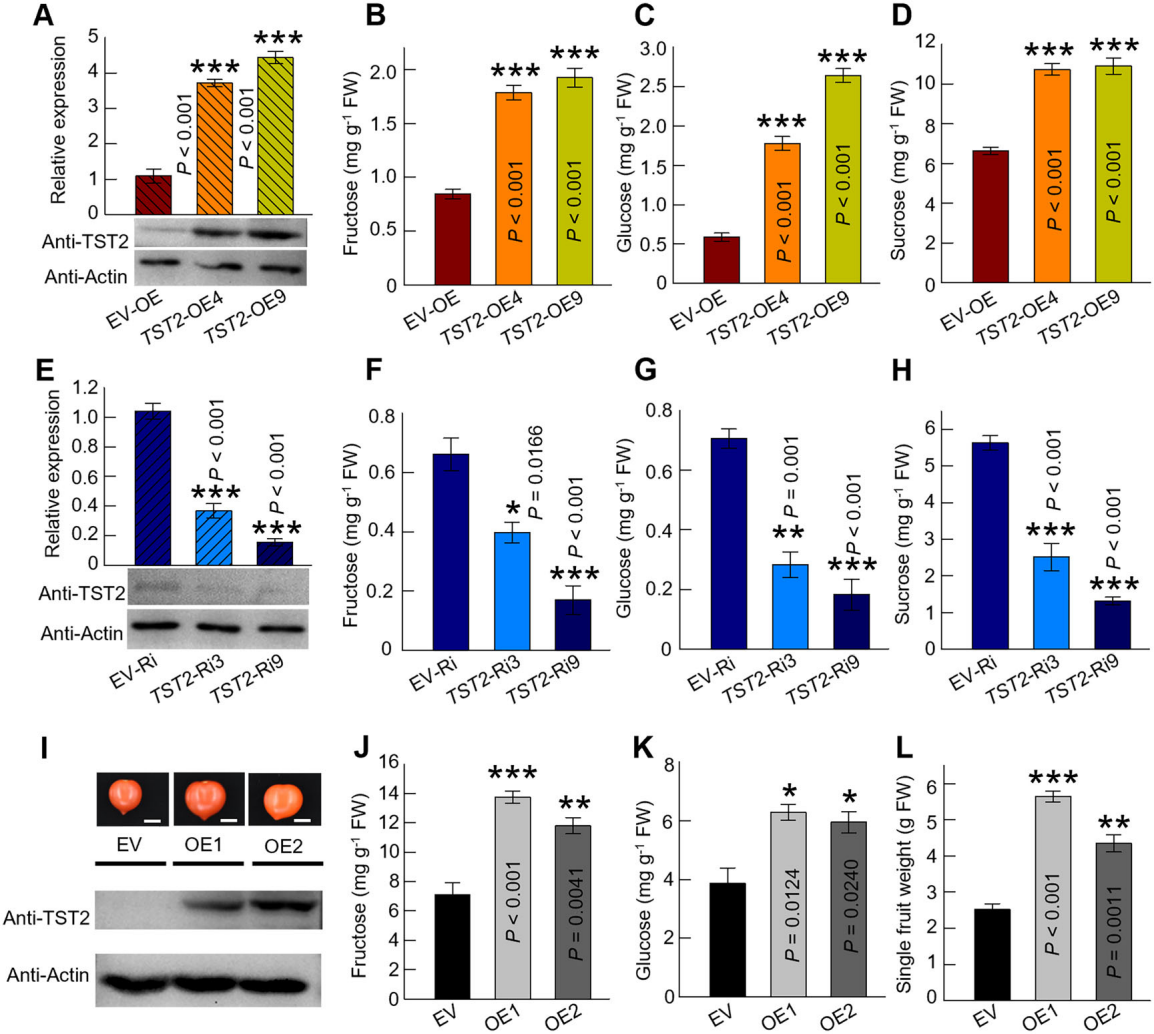
***Citrus sinensis*** **tonoplast‐localized sugar transporter 2 (CsTST2) contributes to the accumulation of sugars** **(A–D)** The transcript (upper) and protein (lower) expression level of CsTST2 **(A)**, and fructose **(B)**, glucose **(C)**, and sucrose **(D)** content assay in overexpression transgenic citrus calli. The overexpression empty vector (EV‐OE) sample works as control. **(E–H)** The transcript (upper) and protein (lower) expression level of CsTST2 **(E)**, and fructose **(F)**, glucose **(G)**, and sucrose **(H)** content assay in RNA interference (RNAi) transgenic citrus calli. The RNAi empty vector (EV‐Ri) sample works as control. **(I)** The fruit photos and the protein levels of CsTST2 in overexpression transgenic tomato lines (OE1 and OE2). Scale bars = 1 cm. **(J–K)** The fructose **(J)** and glucose **(K)** content in EV, and *CsTST2‐*OE tomato fruits. **(L)** The single fruit weight of EV and *CsTST2‐*OE tomato lines (four to eight fruits of each line per replicate). The bars represent the mean value ± *SE* of three independent replicates. *Represents a significant difference from the EV (statistics, one‐way analysis of variance (ANOVA) with Tukey's test, *P < 0.05, **P < 0.01, ***P < 0.001). The protein level of CsTST2 was detected by its specific antibody. β‐actin was used to monitor equal sample loading. FW in **(B–D, F–H, J–K)**: fresh weight.

To assess the function of CsTST2 in sugar accumulation, the overexpression (OE) and RNA interference (RNAi) transgenic citrus calli were generated via *Agrobacterium tumefaciens‐*mediated transformation. Based on the gene expression and protein levels, two OE (*TST2*‐OE4 and OE9) and two RNAi (*TST2*‐Ri3 and Ri9) lines were separately selected for further phenotype assay (Figure 3A, E). As expected, the higher expression of *CsTST2* increased the fructose, glucose and sucrose content in citrus calli (Figure 3B–D), whereas sugar content was decreased with the lower expression level of *CsTST2* (Figure 3F–H). The transient OE of *CsTST2* also induced the sugar accumulation in juice sacs (Figure S5). Additionally, we overexpressed *CsTST2* in tomato and utilized fruits from the T2 generation for the quantification of sugar content. The western blot result by using the CsTST2‐specific antibody confirmed that two stable tomato genetic lines, OE1 and OE2 were established (Figure 3I). The fructose and glucose content were dramatically higher in *CsTST2*‐OE tomato fruit compared to the empty vector (EV) control fruit (Figure 3J, K). But it was different from citrus calli that the sucrose content of the overexpressing *CsTST2* tomato did not increase (Figure S6A). The lack of sucrose sugar source accumulation in tomato fruits may account for this phenomenon (Sun et al., 2022). Further, the transgenic tomato fruit was substantially heavier and bigger than the EV control fruit (Figures 3L, S6B), suggesting that the efficient accumulation of carbon source by the OE of *CsTST2* may lead to larger fruits and higher yield. Taken together, the transgenic phenotypes indicated that CsTST2 positively contributed to sugar accumulation.

### CsCIPK23 interacts with CsTST2 *in vitro* and *in vivo*

**Figure 4:**
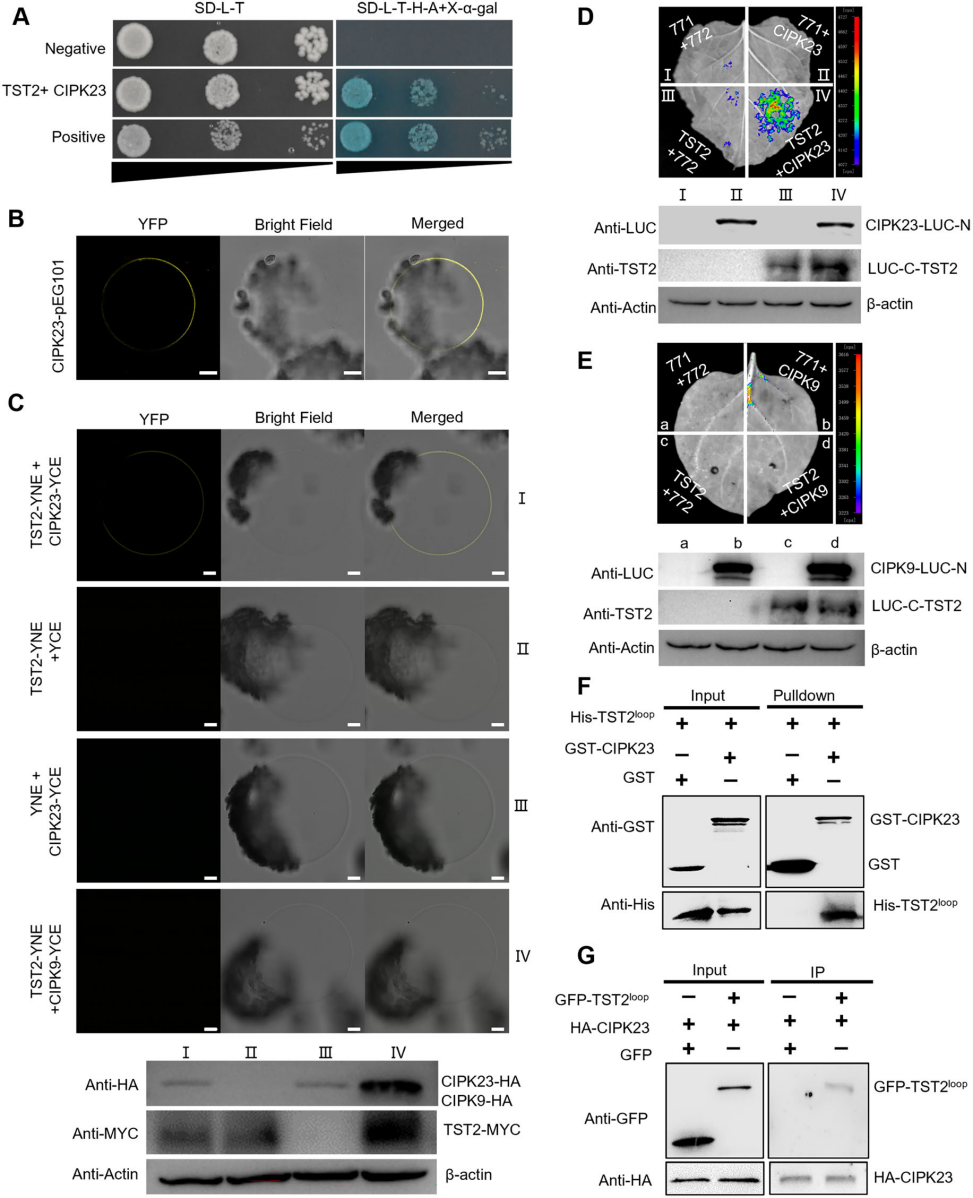
***Citrus sinensis*** **tonoplast‐localized sugar transporter 2 (CsTST2) interacts with** ***C. sinensis*** **calcineurin B‐like protein kinase 23 (CsCIPK23)** **(A)** The interaction analysis between CsTST2 and CsCIPK23 by DUAL membrane yeast system. *CsTST2*‐pBT3‐STE vector and *CsCIPK23*‐pPR3‐N vector were co‐transformed into yeast NMY51 cells, which were grown on synthetic dropout (SD)/‐Leu‐Trp or SD/‐Leu‐Trp‐His‐Ade + X‐α‐gal medium for 3–5 d. Plasmids containing the pNubG‐Fe65/pTSU2*‐*APP and *CsTST2*‐pBT3‐STE/pPR3‐N were used as positive and negative control, respectively. **(B)** The CsCIPK23 protein was localized in the vacuole membrane. Image of confocal microscopy was an isolated vacuole of *Nicotiana benthamiana* leaf expressing CsCIPK23 tagged with yellow fluorescent protein (YFP). Scale bars = 10 μm. **(C)** The bimolecular fluorescence complementation (BiFC) results showing the interaction between CsTST2 and CsCIPK23 in vacuole membrane of *N. benthamiana* leaves. The indicated plasmid vectors were co‐transformed into *N. benthamiana* leaves. Co‐expression of *CsCIPK23*‐YCE/YNE, *CsTST2*‐YNE/YCE, and *CsTST2*‐YNE/*CIPK9*‐YCE were used as negative controls. Scale bars = 10 μm. The protein expression was tested by western blot assay. YNE tagged with MYC, YCE tagged with hemagglutinin (HA). β‐actin was used to monitor equal sample loading. **(D)** Luciferase complementation imaging (LCI) assay of CsTST2 and CsCIPK23 interaction in *N. benthamiana* leaves. The leaves contained different pairs of constructs as indicated. Co‐expression of *CsCIPK23*‐772/771, *CsTST2*‐771/772, and JW771/JW772 were used as negative controls. The protein expression was detected by western blot assay with the indicated antibodies. **(E)** CsCIPK9 was selected as the homologous control to detect the interaction with CsTST2. No fluorescent signal was presented in *CsTST2*‐771/*CIPK9*‐772 combination as well as negative controls. The protein expression was detected by western blot assay with the indicated antibodies. **(F)** *In vitro* pull‐down assay shows the interaction of CsTST2^loop^ with CsCIPK23. CsCIPK23, and CsTST2^loop^ were fused to glutathione S‐transferase (GST) and His, respectively. The purified GST or GST‐CsCIPK23 protein was incubated with His‐TST2^loop^. After immunoprecipitation with GST agarose beads, input and pull‐down samples were detected by western blot with His and GST antibodies. **(G)** Co‐immunoprecipitation (Co‐IP) assay of CsCIPK23‐HA and CsTST2^loop^‐GFP (green fluorescent protein). CsCIPK23‐HA/CsTST2^loop^‐GFP, CsCIPK23‐HA/GFP constructs were expressed in *N. benthamiana* leaves. Proteins were immunoprecipitated with anti‐HA magnetic beads, and were analyzed by HA and GFP antibodies.

To gain insights into the regulation mechanism underlying CsTST2‐induced sugar accumulation, the DUAL membrane yeast two‐hybrid (Y2H) system was applied to screen the interacting proteins of CsTST2. Fourteen members were identified according to the sequence result (Table S1). Given the vital role of calcium in the regulation of fruit quality, the gene annotated as *CBL‐interacting protein kinase 23* (*CIPK23*) was selected for the further analysis. Based on the yeast growth result, CsTST2 was proven to interact with the CsCIPK23 (Figure 4A). Furthermore, to explore the possibility of CSTST2 working with other CIPK family members, a total of 16 members were identified from sweet orange genome and we conducted the necessary point‐to‐point yeast growth analysis (Figure S7). The specific interaction between the CsTST2 and CsCIPK23 protein was discovered (Figure S8). Meanwhile, the expression of *CsCIPK23* was relatively higher and increased with citrus fruit development according to previous RNA sequencing (RNA‐seq) data (Feng et al., 2021) and reverse transcription – quantitative polymerase chain reaction (RT‐qPCR) results (Figures S9, S10), which is similar with the expression pattern of *CsTST2*. Moreover, the presence of CsCIPK23‐YFP fusion signal was observed in the vacuolar membrane (Figure 4B), providing evidence for the plausibility of an interaction between CsCIPK23 and CsTST2 *in planta*. The interaction between CsCIPK23 and CsTST2 was further analyzed by *in vitro* and *in planta* methods. For the luciferase complementation imaging (LCI) and bimolecular fluorescence complementation (BiFC) assays, the luciferase and tonoplast‐localized YFP signals were clearly observed in *in vivo* experiments when CsCIPK23 and CsTST2 were co‐expressed (Figure 4C, D). However, no interaction signals were observed in both the LCI and BiFC assays between CsTST2 and CsCIPK9 (Figure 4C, E), which was selected as the negative control due to its high homology with CsCIPK23 (Figure S7) (Kudla and Bock, 2016). In addition, the non‐transmembrane loop region of CsTST2 (TST2^loop^) was predicted and cloned for glutathione S‐transferase (GST) pull‐down and co‐immunoprecipitation (Co‐IP) analyses (Figure S11). The GST‐tagged CsCIPK23 successfully pulled down the His‐CsTST2^loop^ protein, whereas GST alone did not (Figure 4F). Consistently, the Co‐IP result performed in *Nicotiana benthamiana* leaves confirmed that CsCIPK23 can interact with CsTST2^loop^ *in planta* (Figure 4G). CBL proteins function as Ca^2+^ sensors and regulators by interacting with CIPKs to exert their signaling roles (Luan, 2009). Sixteen CsCBL proteins were discovered and named based on the polygenetic analysis with Arabidopsis CBL family members, and the expression of identified *CsCBLs* in citrus fruit development was also presented according to previous RNA‐seq data (Figures S12, S13). The Y2H result suggested an interaction between CsCIPK23 and CsCBL1 (Figures S14A). This interaction was re‐confirmed by the LCI assay between CsCIPK23 and selected relative higher expression CsCBL members, including CsCBL2/3/7/9/15. The luciferase signal was consistently detected only when CsCIPK23 and CsCBL1 were co‐expressed, while no signal was observed with other combinations (Figure S14B). Further, the localization of CsCBL1 was observed in both the cytoplasmic and nuclear subcellular regions through the utilization of the GFP fusion signal in *N. benthamiana* epidermal cells and isolated protoplasts (Figure S15). Overall, these results support the notion that CsCBL1 is likely responsible for decoding calcium signaling in the cytoplasm, thereby facilitating interaction with CsCIPK23 to regulate the transport activity of CsTST2 and sugar accumulation in citrus.

### CsTST2 is phosphorylated by CsCIPK23

**Figure 5:**
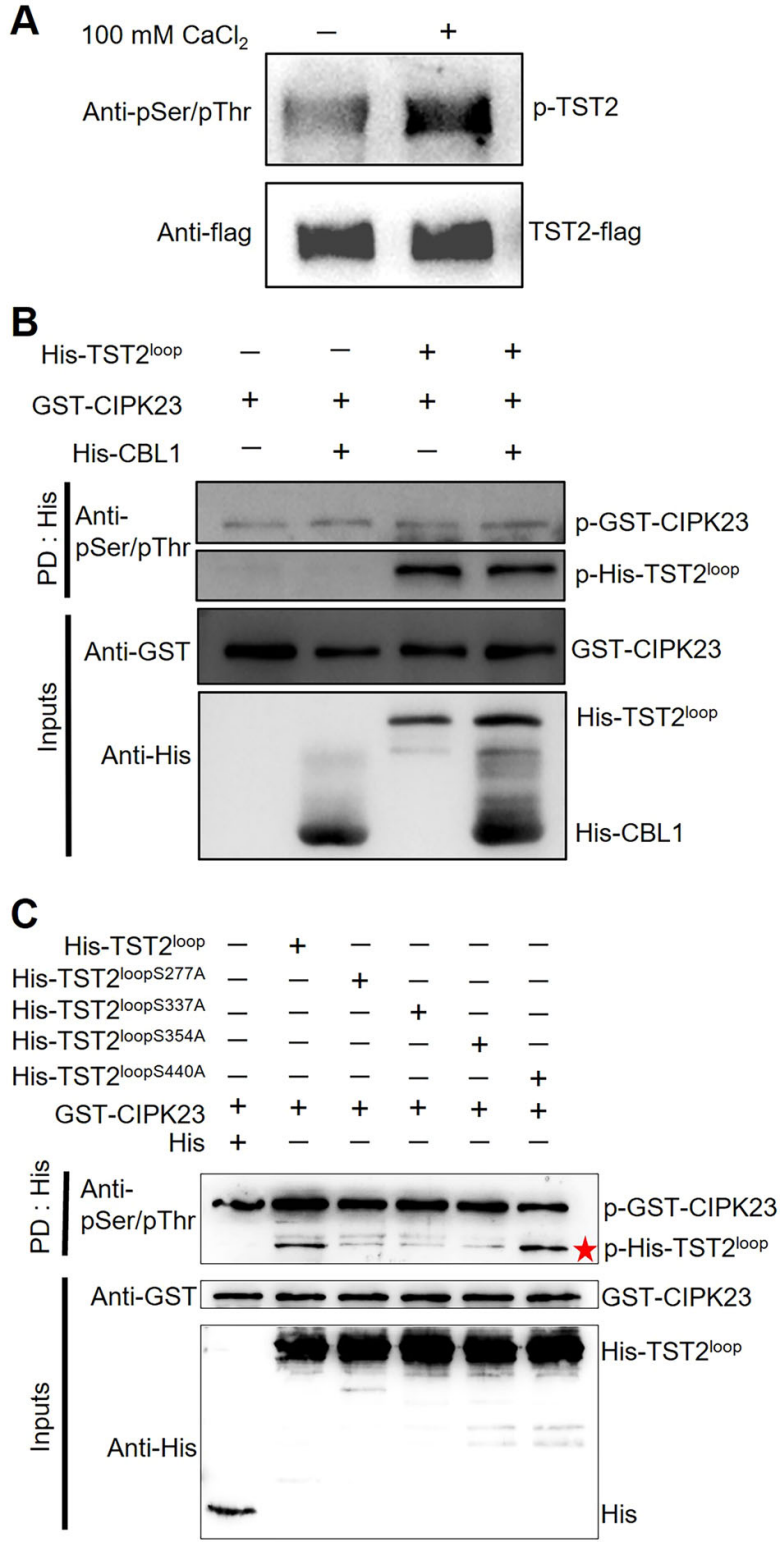
***Citrus sinensis*** **calcineurin B‐like protein kinase 23 (CsCIPK23) phosphorylates** ***C. sinensis*** **tonoplast‐localized sugar transporter 2 (CsTST2) at the Ser277, Ser337, Ser354 residues** **(A)** Ca^2+^ enhances the phosphorylation level of CsTST2 *in vivo*. CsTST2‐overexpressing calli was pre‐treated with 100 mmol/L CaCl_2_ or ddH_2_O for 2 h. The CsTST2‐flag protein of treated calli was immunoprecipitated with anti‐flag magnetic bead, and then immunoblotted with anti‐pSer/pThr and anti‐Flag antibody. **(B)** CsCIPK23 phosphorylates CsTST2 *in vitro*. The phosphorylation reactions were performed using glutathione S‐transferase (GST)‐CIPK23 as the kinase and His‐CsTST2 as the substrate, and proteins containing His‐CsCBL1 with GST‐CIPK23 or only GST‐CIPK23 as negative control. After separation by sodium dodecyl sulfate polyacrylamide gel electrophoresis (SDS‐PAGE), the phosphorylated CsTST2 protein was detected by anti‐pSer/pThr antibody. **(C)** Ser277, Ser337, Ser354 were the phosphorylating sites of CsTST2 by CsCIPK23. The purified GST‐CsCIPK23 or GST was incubated with His‐CsTST2^loop^, and mutant forms included His‐CsTST2^loopSer277^, His‐CsTST2^loopSer337^, His‐CsTST2^loopSer354^, His‐CsTST2^loopSer440^ in kinase reaction buffer for 30 min at 30°C. The proteins were separated by SDS‐PAGE. Anti‐pSer/pThr, anti‐ GST and anti‐His antibodies were used to detect phosphorylating His‐CsTST2^loop^ (p‐His‐CsTST2^loop^), GST‐CsCIPK23 and His‐CsTST2^loop^. Asterisk indicates the bands of phosphorylating His‐CsTST2^loop^ protein.

CsCIPK23 works as a protein kinase that transmits calcium signals by phosphorylating serine (Ser) or threonine (Thr) sites of downstream targets. To figure out this mechanism, we first assessed the changing phosphorylation levels of CsTST2 under calcium conditions. The transgenic calli with the expression of CsTST2‐Flag protein was treated with either (100 mmol/L CaCl_2_ or ddH_2_O for 2 h. Then the CsTST2‐Flag protein was enriched via the Flag beads immunoprecipitation process for the phosphorylation level assay. As shown in Figure 5A, the CsTST2 protein had a relatively higher phosphorylation level under calcium treatment in comparison with the control condition, which demonstrated that calcium induced the phosphorylation modification of CsTST2 *in vivo*. Additionally, *in vitro* phosphorylation kinase assay revealed that CsCIPK23 directly phosphorylated the loop region of CsTST2, with or without CsCBL1 (Figure 5B). Notably, CsCBL1 did not significantly alter CsCIPK23's phosphorylation activity for CsTST2, which was similar to previous reports. For instance, the phosphorylation of manganese transporter MTP8 by CIPK26 was unaffected by CBL2 (Ju et al., 2022). The phosphorylation of salt overly sensitive 1 (SOS1) and ANN4 by CIPK24 remained unaltered in the presence of CBL4 (Hashimoto et al., 2012; Ma et al., 2019). These findings may suggest distinct phosphorylation mechanisms employed by CIPKs on CBLs, at least under *in vitro* experimental conditions (Hashimoto et al., 2012). In consideration of phosphorylation of amino acid is critical to the functional regulation of substrate proteins, we next detected the phosphorylation sites of CsTST2 through a mass spectrometry assay. Accordingly, four candidate phosphorylation sites (Ser277, Ser337, Ser354, and Ser440) by CsCIPK23 were discovered in the loop region of CsTST2 (Figure S16). To determine whether CsCIPK23 can phosphorylate CsTST2^loop^ at these sites, we then individually mutated the identified Ser residues into non‐phosphorylation residues of alanine (Ala). When Ser277, Ser337, and Ser354 were altered into alanine, the phosphorylated bands of CsTST2 were considerably weaker than the un‐mutated ones in the *in vitro* kinase experiment, whereas the bands of the CsTST2^loopSer440A^ mutant remained similar to the CsTST2^loop^ sample (Figure 5C). This result implied that CsTST2 is phosphorylated by CsCIPK23 kinase at Ser277, Ser337, and Ser354 sites.

### The phosphorylation Ser sites are essential for CsTST2 sugar transport activity

**Figure 6:**
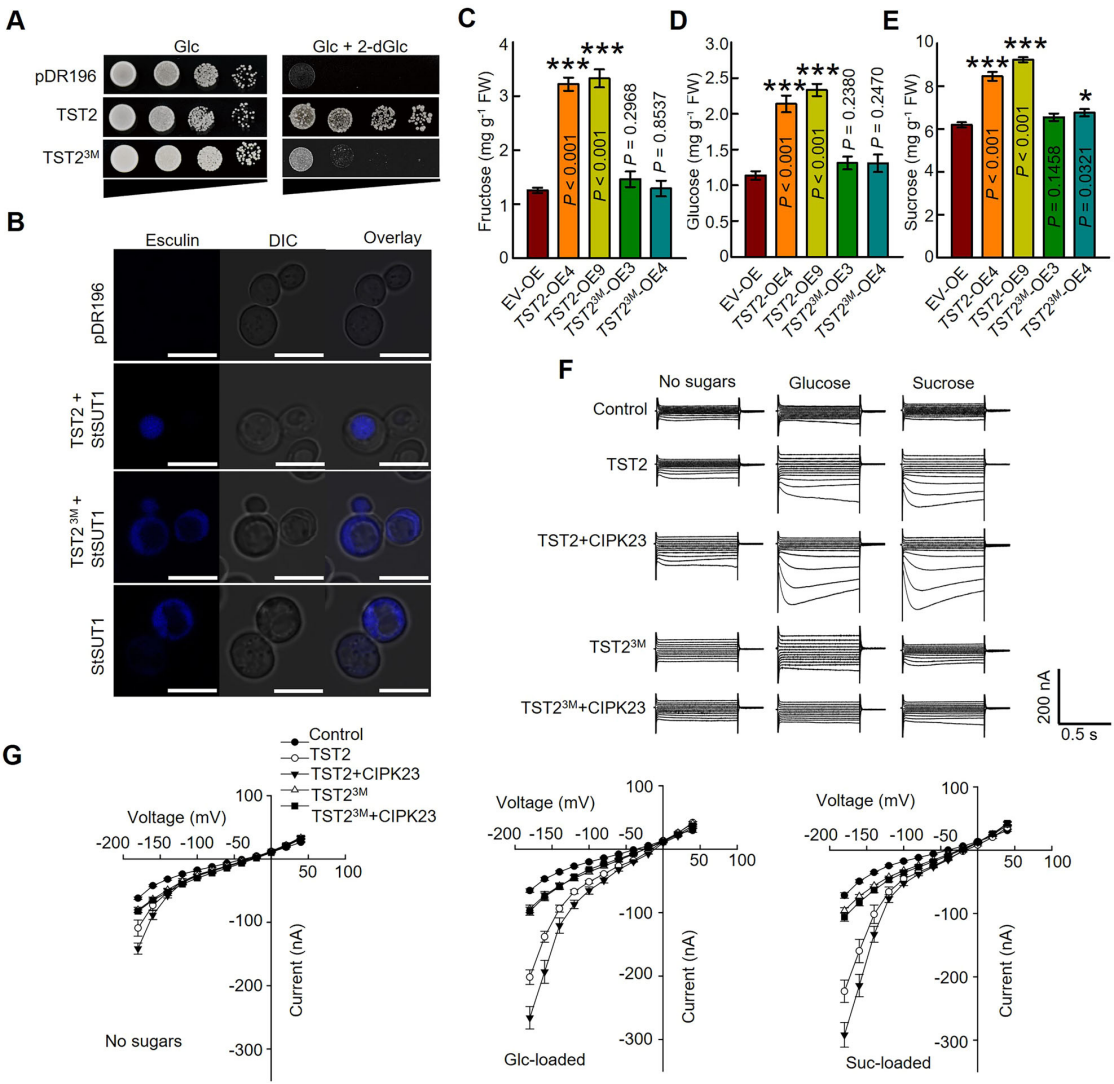
**The phosphorylation residues are essential for the sugar transport activity of** ***Citrus sinensis*** **tonoplast‐localized sugar transporter 2 (CsTST2)** **(A, B)** The transporter activity assay of CsTST2^S277A/337A/354A^ (CsTST2 protein was designed into mutation. Ser277, Ser337, and Ser354 amino acids were mutated to Ala, namely CsTST2^3M^) in yeast system. **(A)** The 2‐dGlc transport and yeast growth assay of CsTST2^3M^. Droplet test with yeast W303 cells containing the empty control, positive control (CsTST2) and construct vector expressing *CsTST2* ^*3M*^. **(B)** The esculin uptake assay of CsTST2^3M^. The esculin signals of co‐expressed *StSUT1* and *CsTST2* ^*3M*^ yeast cells were concentrated in cytosol similar to yeast cells with expression of *StSUT1* alone. The empty control cells have no signal. Scale bars = 5 μm. **(C–E)** The phenotype of *CsTST2* ^*3M*^ transgenic lines (*TST2* ^*3M*^‐OE3 and *TST2* ^*3M*^‐OE4). The fructose **(C)**, glucose **(D)**, and sucrose **(E)** contents were scarcely increased in the *CsTST2* ^*3M*^ overexpressing calli lines, in comparison with empty vector control (EV‐OE). FW: fresh weight. The bars represent the mean value ± *SE* of three biological replicates. Statistical significance was determined using one‐way analysis of variance (ANOVA) with Tukey's test (*P < 0.05, ***P < 0.001). **(F)** Examples of currents elicited by control, *CsTST2* and *CsTST2* ^*3M*^ expressing cells with sugar loaded or not under pH5.5 bath solution, and cells co‐expressing indicated combinations of CsCIPK23, CsTST2, or CsTST2^3M^. **(G)** The current–voltage (*I*/*V*) relationships constructed from current recordings as those shown in **(F)**, for control cells no sugar (*n* = 24), control cells with glucose (*n* = 26), and control cells with sucrose (*n* = 26); cells expressing CsTST2 no sugar (*n* = 9), CsTST2 with glucose (*n* = 14), and CsTST2 with sucrose (*n* = 11); cells expressing CsTST2^3M^ no sugar (*n* = 14), CsTST2^3M^ with glucose (*n* = 12), and CsTST2^3M^ with sucrose (*n* = 23); cells expressing CsTST2/CsCIPK23 no sugar (*n* = 13), CsTST2/CsCIPK23 with glucose (*n* = 16), CsTST2/CsCIPK23 with sucrose (*n* = 10); cells expressing CsTST2^3M^/CsCIPK23 no sugar (*n* = 11), CsTST2^3M^/CsCIPK23 with glucose (*n* = 19), and CsTST2^3M^/CsCIPK23 with sucrose (*n* = 18). All of data are mean ± *SE* with indicated repeat number of cells.

Given the pivotal role of phosphorylation in regulating protein function, we conducted further analysis to decipher the impact of phosphorylation sites on TST2 transport activity. To this end, we constructed the triple sites mutant, including S277A, S337A and S354A in the CsTST2 loop region, which was named as CsTST2^3M^ for our next research. The localization assay demonstrated that the site mutation of CsTST2^3M^ employed in this study did not alter the protein's subcellular localization or protein stability (Figure S17). Based on the 2‐dGlc transport assay, we found that yeast with the expression of the *CsTST2* ^*3M*^ failed to grow in the 2‐dGlc medium, implying that the CsTST2^3M^ cannot transport 2‐dGlc into the vacuole (Figure 6A). Meanwhile, different from the vacuolar signal as shown in *CsTST2* + *StSUT1* expression yeast, the esculin fluorescence signal was mostly located in the cytoplasmic region with the co‐expression of *CsTST2* ^*3M*^ and *StSUT1* (Figure 6B). Then the *CsTST2* ^*3M*^ OE transgenic calli were generated for the sugar content assay. The *CsTST2* ^*3M*^ failed to induce the fructose, glucose and sucrose content in comparison with TST2‐OE and the control calli, even with the higher transcript level (Figures 6C–E, S18). The collective findings suggest that the mutation of phosphorylation sites in CsTST2 results in a loss or reduction of transport activity and sugar accumulation function in the vacuole.

The significant impact of phosphorylation sites on the function of CsTST2 prompts an inquiry into how CsCIPK23 regulates its transport activity. For this purpose, the cRNA of *CsCIPK23* and *CsTST2* were co‐injected into the *X. laevis* oocyte cells for the TEVC and current recoding, which can reflect the alteration in the transport activity. In the presence of CsCIPK23, larger currents were recorded in cells loaded with sucrose or glucose in comparison with CsTST2 alone (Figure 6F), suggesting that CsCIPK23 can activate the sugar transport activity of CsTST2. Furthermore, the weaker currents were recorded in the cells with the expression of CsTST2^3M^ under sugar‐loaded conditions, whether CsCIPK23 exists or not (Figure 6G). These results demonstrate that CsCIPK23‐mediated phosphorylation is essential for CsTST2's function in sugar transport.

### CsCIPK23‐induced sugar accumulation depends on CsTST2

**Figure 7:**
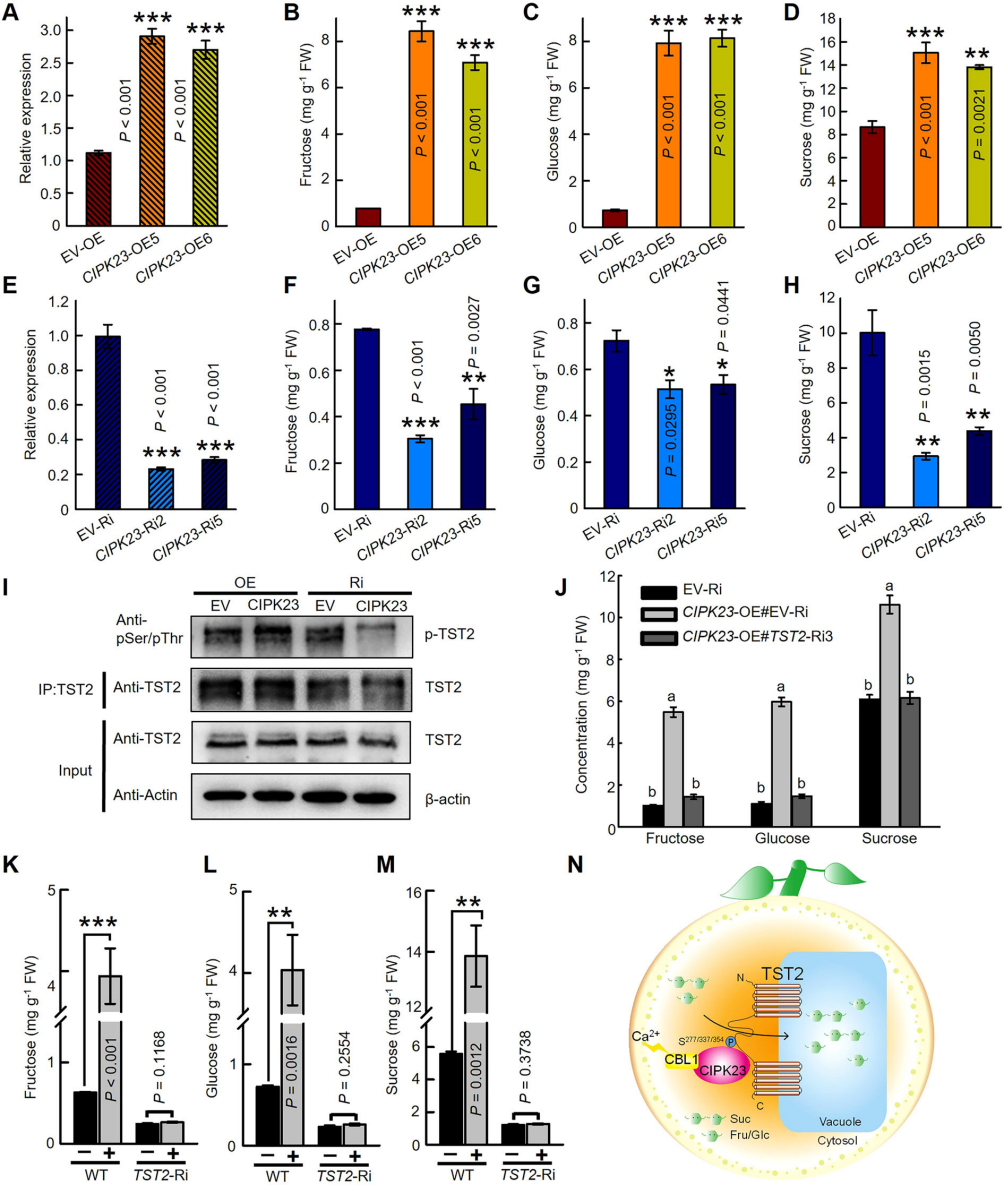
***Citrus sinensis*** **calcineurin B‐like protein kinase 23 (CsCIPK23) induced the sugar accumulation depends on the phosphorylation level of** ***C. sinensis*** **tonoplast‐localized sugar transporter 2 (CsTST2)** **(A–D)** The expression level of *CsCIPK23* **(A)** and fructose **(B)**, glucose **(C)**, and sucrose **(D)** content assay in *CsCIPK23* overexpression transgenic citrus calli. The expression level of *CsCIPK23* **(E)** and fructose **(F)**, glucose **(G)**, and sucrose **(H)** content assay in *CsCIPK23* RNA interference (RNAi) transgenic citrus calli. The bars represent the mean value ± *SE* of three biological replicates. A significant difference from the empty vector (EV) control (statistics, one‐way analysis of variance (ANOVA) with Tukey's test, *P < 0.05, **P < 0.01, ***P < 0.001). **(I)** The phosphorylation level of CsTST2 in CsCIPK23 and empty vector (EV‐OE, EV‐RNAi) transgenic calli. β‐actin was used as an input control. **(J)** The sugar content assay within the overexpression of *CsCIPK23* in control (EV‐Ri) or *CsTST2*‐Ri3 sample. Bars represent the mean value ± *SE* of three biological replicates. Different letters indicate significant differences as assessed by one‐way ANOVA (with Tukey's test), *P* < 0.05. **(K–M)** The fructose **(K)**, glucose **(L)**, and sucrose **(M)** content in wild type (WT) and *CsTST2*‐Ri calli with calcium treatment or not. WT and CsTST2‐RNAi calli were harvested after 15 d treated with 15 mmol/L CaCl_2_. Bars represent the mean value ± *SE* of three biological replicates. Statistics analysis, the asterisks indicate significant differences as assessed by independent samples *t*‐test, **P < 0.01, ***P < 0.001. FW in **(B–D, F–H, J, K–M)**: fresh weight. **(N)** The working model of CsCBL1/CIPK23‐CsTST2 regulates sugar accumulation in citrus vacuole.

To analyze the role of CsCIPK23 in regulation of sugar content, the *CsCIPK23* OE and RNAi transgenic citrus calli were generated and selected based on the transcript levels (Figure 7A, E). The higher contents of fructose, glucose and sucrose were detected within the *CsCIPK23*‐OE transgenic lines (*CIPK23*‐OE5, OE6), whereas the lower expression of *CsCIPK23* decreased the sugar content in the RNAi lines (*CIPK23*‐Ri2, Ri5) (Figure 7B–D, F–H). This phenotype implied that CsCIPK23 positively regulates sugar accumulation. Nevertheless, it remained unclear what the role of CsTST2 in CsCIPK23‐induced sugar accumulation process is, as they have been shown to interact with each other (Figure 4). To further elucidate if CIPK23‐mediated increase of sugar content requires phosphorylation of CsTST2, an *in vivo* phosphorylation assay was conducted. The CsTST2 protein was enriched from the CsCIPK23‐OE, RNAi, and empty vector transgenic calli using the anti‐TST2 antibody immunoprecipitation method for the phosphorylation level assay. Notably, high phosphorylation levels of CsTST2 were observed in *CsCIPK23*‐OE lines, whereas the relatively weak phosphorylation bands were detected in *CsCIPK23*‐RNAi lines compared with its EV control and *CsCIPK23*‐OE samples within similar amounts of CsTST2 protein (Figure 7I). The results demonstrated that the alteration of CsCIPK23 expression affects the phosphorylation level and transport activity of CsTST2, but not protein stability. Furthermore, citrus calli co‐transformation was employed to elucidate the genetic link between CsCIPK23 and CsTST2. The sugar content was increased significantly with the OE of *CsCIPK23* in EV control calli (EV‐Ri). However, the accumulation of sugars was partially disrupted when *CsCIPK23* was overexpressed in the *CsTST2*‐RNAi background calli (Figures 7J, S19). Consistently, calcium treatment induced the higher sugar content in wild type (WT) calli, but failed in the *CsTST2*‐RNAi line (Figure 7K–M). Taken together, these results unequivocally defined that calcium or CIPK23‐mediated sugar accumulation is dependent on CsTST2 and its phosphorylation level (Figure 7N).

## DISCUSSION

Sugar content establishes fruit quality and sweetness, which is commonly quantified as the total soluble solid (Katuuramu et al., 2023). Recently, we obtained proteomic data on the vacuolar composition of citrus fruit and identified candidate proteins involved in vacuolar sugar accumulation through correlation analysis of protein expression and sugar content (Mao et al., 2024). In this study, we provide evidence that CsTST2 exhibits sucrose and hexose transport activity and predominantly contributes to sugar accumulation in citrus fruit. Further investigations revealed that the calcium signal response protein complex CsCBL1/CIPK23 phosphorylates CsTST2 to enhance its transport activity and sugar content.

Sugar transporters have always been the focus of fruit quality research. These proteins exhibit distinct transmembrane forms and spatial localization, thus enabling the transport of various sugar substrates (Matsui et al., 2017; Gill et al., 2021; Zhang et al., 2021a, 2021b). Here, we demonstrated that tonoplast‐localized CsTST2 has sucrose and hexose transport activity through two independent yeast and oocyte systems (1, 2). The genetic transformation results showed that OE of *CsTST2* positively increased sugar content, while interference with its expression decreased sugar accumulation (Figure 3). These findings reveal a close relationship between the transport function of CsTST2 and the soluble sugar accumulation in citrus fruit. However, the members of TST in other species were reported to have different transport substrates, including monosaccharides, sucrose, or both of them. For example, BvTST2.1 was identified as a sucrose‐specific carrier protein in sugar beet taproots by using electrophysiological assay and transgenic Arabidopsis mutants (Jung et al., 2015). The OE of *GhTST2* in cotton plants induced high content of glucose, but not sucrose, implying the hexose transport characteristic of GhTST2 (Deng et al., 2020). More recently, MdTST1/2 was reported to be responsible for both sucrose and hexose accumulation in apple and tomato (Zhu et al., 2020, 2023). The observed substrate discrepancy of TSTs could potentially arise from variations in metabolic processes across different varieties, resulting in the accumulation of distinct sugars. Alternatively, the evolutionary variations in protein sequence and structure may account for the diverse substrate transport mechanisms. Thus, the more intricate transport function of TSTs, encompassing transport activity and substrate selectivity, deserves further investigation in terms of potential sequence and structural disparities.

Phosphorylation is one of the important regulation mechanisms for membrane transporter activity. We screened the interacting protein CsCIPK23 of CsTST2 by DUAL membrane Y2H system (Figure 4A). The BiFC, LCI, GST pull‐down and Co‐IP results further clarified that CsCIPK23 can interact with the cytoplasmic loop region of CsTST2 (Figure 4C–G). Additionally, the CIPK family comprises multiple members and it has been reported that several of these members are capable of simultaneous interaction with target protein (Ju et al., 2022). In order to validate the interactions among other CsCIPK family members, we conducted Y2H analysis on a total of 16 candidates (Figure S7). The findings revealed that solely CsCIPK23 and CsTST2 exhibited interaction (Figure S8). Meanwhile, CsCIPK23 and CsTST2 exhibit similar transcriptional expression patterns, characterized by high expression levels in fruits that progressively increase during fruit maturation (Figures 1, S9, S10). The fusion signal of CsCIPK23‐YFP was also observed in the vacuolar membrane (Figure 4B). This result further reinforces the confidence in the interaction between CsCIPK23 and CsTST2. Moreover, CsCBL1 was identified to interact with CsCIPK23 to make a calcium signal transduction complex (Figures S13, S14). In contrast to previous finding, despite harboring an MGXXX(S/T) consensus sequence for N‐myristoylation, CsCBL1 has been demonstrated to localize in the cytoplasm and nucleus rather than the cellular membrane (Figure S15). This suggests that while structurally divergent N‐terminal domains of CBL proteins are important, they do not exclusively determine their targeting patterns (Batistic et al., 2008; Batistic et al., 2010). The findings imply that CsCBL1 in citrus may possess distinct localization signals compared to other reported CBL1 proteins, such as those found in the cytoplasm and nucleus. It has been reported that CBLx/CIPK23 emerged as a central regulator governing root responses to various environmental stresses via interacting with various plasma membrane or vacuole membrane transporters in plants (Ródenas and Vert, 2021). Our study has expanded the new functional repertoire of CsCIPK23, which is involved in regulating fruit quality by phosphorylating the tonoplast‐localized sugar transporter. In conclusion, the CsCBL1/CIPK23 module serves as a potential mechanism for regulating CsTST2 transport function and sweetness in response to calcium signals in sweet orange.

Phosphorylated serine or threonine sites usually play vital roles in protein function. To elucidate the molecular mechanism underlying CsCIPK23 regulation of CsTST2, we conducted liquid chromatography – mass spectrometry (LC‐MS) to identify the specific phosphorylation sites on TST2 targeted by CsCIPK23 (Figure S16). Subsequently, through *in vitro* phosphorylation analysis and point mutation experiment, we confirmed that serine residues at positions 277th, 337th, and 354th of the CsTST2 loop region were phosphorylated by CsCIPK23 (Figure 5). The transport activity of non‐phosphorylated residue (alanine) mutant CsTST2^3M^ was dramatically inhibited, and the sugars failed to increase with the OE of *CsTST2* ^*3M*^ in transgenic lines (Figure 6). In addition, we also observed that CsCIPK23 can facilitate sugar accumulation, and this process was contingent upon the presence of CsTST2 and its phosphorylation level (Figure 7). Moreover, calcium‐induced sugar accumulation was accompanied by increased phosphorylation of CsTST2 (Figures 5A, 7K–M). The above contents strongly indicate that calcium may function as a signaling molecule to modulate the phosphorylation of CsTST2 through the CsCBL1/CIPK23 complex, thereby ultimately impacting the accumulation of soluble sugars in the citrus fruit vacuole.

According to previous reports, TST protein members were identified to interact with distinct kinase proteins in different species. For instance, GhCBL2/CIPK6 interacted with GhTST2 to improve sugar contents and abiotic stress resistance in cotton plant (Deng et al., 2020). CPK28 was reported to regulate sugar content through interaction with TST4 in pear (Li et al., 2023a). The latest report indicated that protein kinase CPK27 phosphorylated TST2 to promote sugar accumulation under drought stress in tomato plant (Zhu et al., 2024). By sequence alignment, we found that the conserved 277^th^ serine residue of TSTs in citrus, pear and Arabidopsis were all phosphorylated, indicating that this site is conservative and critical for the function of TST proteins. However, Ser337 and Ser354 sites in CsTST2 modified by CsCIPK23 were newly identified phosphorylation sites in comparison with the other species’ TST members (Figure S20). The results indicate that the phosphorylation modification of TSTs by calcium sensor proteins exhibits not only certain conserved properties, but also distinct individual variations in different species. In summary, our study has demonstrated the crucial role of Ser277, Ser337, and Ser354 sites in the transport function of CsTST2, thereby expanding our understanding of the molecular mechanism underlying calcium signaling proteins in regulation of vacuolar membrane sugar transporter and the sweetness quality of citrus fruit.

## MATERIALS AND METHODS

### Plant materials and growth conditions

“Anliu” sweet orange (*Citrus sinensis* cv. Anliu) from Huazhong Agricultural University orchard was used in this study. Fruits were sampled at 90, 120, 150, 180, 210, and 240 day after flowering (DAF) for sugar content and gene expression assay. Citrus calli were grown on Murashige and Tucker (MT) medium at 25°C in the dark for stable transformation. Tomato (*Solanum lycopersicum L*. Micro Tom) and *N. benthamiana* seeds were germinated on Murashige and Skoog (MS) medium (Coolaber, Beijing, China) and the seedlings were grown in a growth chamber at 24°C with 65% relative humidity under a 14‐h light photoperiod.

### Phylogenetic analysis

Amino acid sequences of previously reported TST members in *Arabidopsis thaliana*, apple (*Malus domestica*), grape (*Vitis vinifera*), melon (*Cucumis melo*) and watermelon (*C. lanatus*) and selected query peptide sequences in *C. sinensis* were put into MEGAX64 software. The sequences were aligned using the ClustalW method with default parameters and a phylogenetic tree was constructed using the maximum‐likelihood strategy. The two TST members of *Citrus sinensis* were named as CsTST1 and CsTST2 according to the structure of the tree and their relationship with Arabidopsis (Zhao et al., 2022). The phylogenetic analysis of CsCBLs and CsCIPKs was the same as decribed above.

### RNA extraction, cDNA synthesis, RT‐qPCR analysis

The EASYspin Plus Plant RNA Kit (RN38; Aidlab, Beijing, China) was utilized for the extraction of total RNA. One microgram of total RNA from each sample was employed to generate first‐strand cDNA using the TransScript® IV One‐Step gDNA Removal and cDNA Synthesis SuperMix (TransGen, Beijing, China). The cDNA derived from Anliu fruits served as a template for cloning genes of *CsTST2, CsCIPKs* and *CsCBLs*. For RT‐qPCR experiments, ChamQ Universal SYBR qPCR Master Mix (Vazyme, Nanjing, China) was used to analyze the transcript levels of *CsTSTs* and *CsCIPK23* from Anliu fruits and transgenic citrus calli. Primers used in this study are listed in Table S2.

### Protein extraction and immunoblot analysis

Proteins were extracted from Anliu fruit at the indicated time and quantified as described by Zhu et al (2023). For immunoprecipitation analysis of CsTST2, the TST2 antibody (generated against the peptide IEKDMVPPAHGTLSSMRHGSQVQGNAGEPVGM from 370th aa to 401th aa of CsTST2 protein sequence in rabbit by Abmart, Shanghai, China) was combined with magnetic beads (BeyoMagTM Protein A + G; Beyotime Biotechnology, Shanghai, China) for 2 h at 4°C to form a complex, which was then incubated with the total protein of measured samples for another 4 h. For immunoblot analysis, the extracted protein was incubated with the primary CsTST2 antibody after a 3,000‐fold dilution with blocking buffer, and then with secondary antibody (Goat Anti‐Rabbit Immunoglobulin G‐Horseradish Peroxidase, M21002; Abmart, Shanghai, China) after a 3,000‐fold dilution. The FemtoLight ECL Substrate and Oxidant mix solution (SQ201; Epizyme Biotech, Shanghai, China) was added to the blot for 1–2 min until the desired color developed. *β*‐actin (Anti‐*β*‐Actin polyclonal antibody, BL005B; Biosharp, Hefei, China) was used to confirm equal sample loading.

### Subcellular localization

The full length coding sequence (CDS) of *CsTST2* (2,199 bp) without a stop codon was amplified from sweet orange and cloned into the pRI101‐GFP vector with *35S* promoter for subcellular localization assay. In addition, the full length CDS of *CsCIPK23* (1,395 bp) and *CsCBL1* (642 bp) without stop codons were constructed into the pEYFP101 vector and pRI101‐GFP vector, respectively. the CsCBL1‐GFP was co‐expressed with plasma membrane marker protein of AtCBL1‐OFP (oraange fluorescent protein) in *N. benthamiana* leaves. These fluorescent signals were observed under confocal microscope (TCS SP8; Leica, Mannheim, Germany) after over 48 h of the *A. tumefaciens*‐mediated transient infiltration of *N. benthamiana* leaves. To detect the tonoplast signal, protoplast isolation and vacuole release were performed according to the previous report (Li et al., 2020b). The YFP signal was triggered at 514 nm and detected with a 520–540‐nm band‐pass filter. GFP was excited at 488 nm and emitted a signal detected using a 500–540‐nm band‐pass filter. Furthermore, the plasma membrane marker (Batistic et al., 2008) and chlorophyll autofluorescence signal excitation wavelength was 552 nm, while the emission signal was observed between 590 and 650 nm. Primers used in this study are listed in Table S2.

### Functional analysis of CsTST2 by heterologous expression in yeast

The full length CDS of *CsTST2* was amplified using Phusion polymerase (TransGen, Beijing, China) with gene‐specific primers CsTST2‐PYGFP‐F and CsTST2‐PYGFP‐R in Table S2. The sequence was cloned into the pYES‐DEST2‐eGFP vector with the *ADH1* promoter for expression and subcellular localization assay in yeast strain with the GFP fluorescence under confocal microscope (TCS SP8; Leica, Mannheim, Germany). To determine the sugar transport properties, the CDS of *CsTST2* or its site mutant (*CsTST2* ^*3M*^) was inserted into the pDR196 vector and transferred into the yeast strain W303 (*MATa/MATα {leu2‐3,112 trp1‐1 can1‐100 ura3‐1 ade2‐1 his3‐11,15} [phi*+*]*). The transformed yeast was grown on synthetic dripout (SD)‐Ura (2% Glc) medium for 48 h at 30°C. Then, yeast droplet tests with an initial optical density at 600 nm (OD_600_) of 0.6 were conducted by using yeast peptone dextrose agar solid medium supplemented with 1% (w/v) Glc and 1% (w/v) Glc + 0.2% (w/v) 2‐dGlc for vacuolar hexose transport assay. For the sucrose transport assay, pDR196‐*CsTST2* or pDR196‐*CsTST2* ^*3M*^ was transformed into the yeast strain W303 that harbored pDR196‐*StSUT1*, which can uptake the sucrose or its analog esculin into the yeast cytosol. The esculin transport assay was performed as previously reported with slightlmodification (Gora et al., 2012). Transformed yeast was cultivated in SD‐Ura (2% Glc) medium until reaching the logarithmic phase (OD_600_ = 0.6–0.8) and then collected after being washed three times with 25 mmol/L phosphate‐buffered saline (PBS) (at pH 5). The yeast pellets were then incubated with 1 mmol/L esculin at 30°C for 2 h with an initial OD_600_ of 0.5 in PBS (at pH 5) buffer. The position and intensity of the esculin signal were detected at 440 nm using a confocal laser‐scanning microscope (TCS SP8, Leica, Mannheim, Germany) after excitation at 405 nm.

### Transformation of calli, tomato plants

The full‐length sequences of *CsTST2*, *CsTST2* ^*3M*^, and *CsCIPK23* without stop codon were amplified by PCR using specific primers containing *Sal*Ⅰ and *Kpn*I restriction sites as shown in Table S2. The resulting amplicon was purified and inserted into the pENTR1A vector at the designated restriction sites through the ClonExpress II one‐step cloning kit (Vazyme, Nanjing, China). Subsequently, the gene was transferred to the pK7WG2D vector with a *35S* promoter utilizing LR Clonase of Gateway technology (Thermo Fisher, Waltham, MA, USA), resulting in the final OE construct. In order to obtain the RNAi vector, a 250 bp of *CsTST2* or *CsCIPK23* fragments was constructed into the pDONR207 vector, and then transferred to the pGWB‐RNAi vector with a *35S* promoter via Gateway technology (Thermo Fisher, Waltham, MA, USA). The OE or RNAi vector was respectively introduced into *A. tumefaciens* EHA105 (Weidi, Shanghai, China), which was utilized for subsequent transformation experiments. The calli cells of suspension culture were drained on filter paper and then placed in MT‐sucrose liquid medium containing EHA105 strain with different constructs under an OD_600_ of 0.6. The transformation through vacuum treatment was referred to in a previous report (Moniruzzaman et al., 2021). Positive calli were screened by antibiotics and RT‐qPCR assay. The *Micro Tom* tomato seeds were disinfected, then were germinated and cultivated in MS basal salt medium. Seven‐d‐old cotyledons were cut in half, and the abaxial side of the cotyledons was incubated on 90 cm glass plates with MS medium (containing 2% sucrose, 0.8% agar supplemented with 2 mg/L zeatin and 0.5 mg/L indoleacetic acid) for *A. tumefaciens‐*mediated transformation as previously reported (Yaroshko et al., 2023). Cultivation conditions were 16 h light and 8 h dark period at 23–25°C for all experiments. The positive transgenic plants were identified by antibiotic screening and protein level assay, from which two positive lines were selected for further studies. Primers used in this study are listed in Table S2.

### DUAL membrane system and Y2H assay

The DUAL membrane system was applied for membrane protein interaction assay. The CDS of *CsTST2* was cloned into the pBT3‐STE vector and *CsCIPK23* was cloned into the pPR3‐N vector from the DUALmembrane starter kit (Ouyi Biomedical Technology, Shanghai, China) as described in the user manual. Various combinations were transformed into the yeast strain NMY51, and positive transformed colonies were selected on SD‐Trp‐Leu‐His‐Ade medium for 5 d at 30°C. The pNubG‐Fe65/pTSU2‐APP and *CsTST2*‐pBT3‐STE/pPR3‐N were, respectively, used as the positive and negative controls. Droplet test was performed in SD‐Trp‐Leu and SD‐Trp‐Leu‐His‐Ade medium containing 40 μg/mL X‐α‐gal (Straub et al., 2017). The regular Y2H method was conducted as previously published (Song et al., 2022). In brief, *CsCBLs* was cloned into pGADT7 (AD) vector and the cDNA of *CsCIPK23* was introduced into the pGBKT7 (BD) vector. The AD and BD constructs were co‐transformed into the Y2H‐Gold yeast strain. Serial dilution of transformed colonies were dropped onto SD‐Leu‐Trp and SD‐Leu‐Trp‐His‐Ade selection media supplemented with 40 μg/mL X‐α‐gal. The protein–protein interaction was assessed by observing of the yeast growth. Primers used in this study are listed in Table S2.

### Glutathione S‐transferase pull‐down assay

For pull‐down assay, the loop region of CsTST2 sequence (from 570 to 1,560 bp) was cloned and inserted into the pET32a construct. The GST‐CsCIPK23 (within pGEX‐4T‐1), His‐CsTST2^loop^, GST recombinant proteins were expressed in *Escherichia coli* Rossetta DE3 strain (Weidi) and purified using Ni‐NTA Purification System and glutathione sepharose, respectively. For the pull‐down assay, the His‐CsTST2^loop^ protein was incubated with GST‐CsCIPK23 or GST protein at 4°C for 4 h, and then incubated with glutathione‐sepharose resin for another 4 h. After elution using binding buffer from the resin, the proteins were subjected to immunoblot analysis with anti‐His (Beyotime Biotechnology, Shanghai, China) and anti‐GST (Beyotime Biotechnology, Shanghai, China) antibodies. Primers used in this study are listed in Table S2.

### Bimolecular fluorescence complementation assay

According to methods previously described (Zhang et al., 2023), the full length CDS of *CsTST2* and *CsCIPK23* or *CsCIPK9* (1329 bp) without stop codon were respectively cloned into the pYAC‐PYNE and pYAC‐PYCE vectors. All constructs were transiently expressed in tobacco leaves by *A. tumefaciens*‐mediated transformation for over 48 h. Confocal microscopy of the released vacuole was performed with a Leica microscope (SP8, Leica, Mannheim, Germany). The YFP signal was excited at 514 nm and emitted a signal detected using a 520 to 540 nm band‐pass filter. Western blot was performed with hemagglutinin (HA), MYC, and *β*‐Actin polyclonal antibodies (Beyotime Biotechnology, Shanghai, China) for protein expression assay. Primers used in this study are listed in Table S2.

### Luciferase complementation imaging assay

The full length CDS of *CsTST2* or *CsCBL1/2/3/7/9/15* was cloned into the JW771‐LUC‐C vector, and *CsCIPK23* was inserted into the JW772‐LUC‐N vector. Next, these constructs were transfected into *A. tumefaciens* cells and combined in various pairings (*CsTST2*‐JW771 and *CsCIPK23*‐JW772, *CsCBL1*‐JW771 and Cs*CIPK23*‐JW772 were designated as the experimental group, *CsCIPK9*‐JW772 and *CsTMT2*‐JW771, *CsCBL2/3/7/9/15*‐JW771 and *CsCIPK23*‐JW772 were used as negative controls). Each combination with a final optical density at OD_600_ of 1.0 in the transformation buffer (10 mmol/L 2‐[*N*‐morpholino]ethanesulfonic acid (MES) pH 5.6, 10 mmol/L MgCl_2_ and 150 μmol/L acetosyringone) was infiltrated into *N. benthamiana* leaves. Western blot was performed with a firefly luciferase antibody (Abmart, Shanghai, China), *β*‐Actin polyclonal antibody and CsTST2 antibody for protein expression assay. Primers used in this study are listed in Table S2.

### Co‐IP assay

For Co‐IP assay, *A. tumefaciens* cells containing *CsCIPK23*‐pGWB414+*CsTST2* ^*loop*^‐pRI101‐GFP or *CsCIPK23*‐pGWB414 + pRI101‐GFP (with *35S* promoter) were infiltrated into *N. benthamiana* leaves and co‐expressed for a duration of 2 d. The total proteins were extracted using plant protein extraction buffer (Beyotime Biotechnology, Shanghai, China). The HA‐beads were added to the supernatant of total protein and incubated together overnight at 4°C, and then the beads were washed four times with 1× Tris‐buffered saline (pH 7.4). Finally, the beads were eluted with 1× sodium dodecyl sulfate (SDS) loading buffer and placed in a 95°C water bath for 5 min. Eluted proteins are used for immunoprecipitation analysis with GFP and HA antibodies (Beyotime Biotechnology, Shanghai, China). Primers used in this study are listed in Table S2.

### Liquid chromatography with tandem mass spectrometry assay

The total protein was extracted from citrus calli with the overexpressing of CsCIPK23‐HA and CsTST2‐Flag via incubation with Flag‐beads at 4°C for 12 h. The SDS polyacrylamide gel electrophoresis (PAGE) was performed to cut off the tape corresponding to the size of the target protein. The sol was then subjected to a series of operations such as protein enzymolysis, salt removal, concentration and drying, and peptide resolubility for the subsequent liquid chromatography with tandem mass spectrometry (LC‐MS/MS) analysis (Thermo Scientific Q‐Exactive HF‐X, Easy‐nLC1200). The mass spectrum database retrieval software is MaxQuant 2.0.1.0 via using the uniprot‐Sweet orange (*Citrus sinensis*) protein database (https://www.uniprot.org/taxonomy/2711). After mass spectrometry data retrieval, peptide‐spectrum match false discovery rate (FDR) ≤0.01, site FDR ≤0.01, and protein FDR ≤0.01 were used as the screening criteria for peptide, modification site and protein identification, respectively. Modified peptide score ≥40 and unmodified peptides ≥0 were added as screening criteria for modified peptide to ensure the identification quality.

### *In vitro* and *in vivo* phosphorylation assays

For *in vitro* assay, the phosphorylation reactions were performed in 60 μL of kinase buffer (25 mmol/L Tris‐HCl, pH 7.5, 10 mmol/L MgCl_2_, 0.1 mmol/L CaCl_2_, 1 mmol/L dithiothreitol, and 4 mmol/L adenosine triphosphate) containing GST‐CsCIPK23, His‐CBL1 and substrate proteins (His‐CsTST2^loop^, His‐ CsTST2^loops277A^, His‐CsTST2^loops337A^, His‐CsTST2^loops354A^, His‐CsTST2^loops440A^) at 29°C with 30 min of gentle shaking. For *in vivo* phosphorylation assay, the protein was extracted from transgenic calli with the expression of CsTST2‐Flag protein with IP buffer containing protease and phosphatase inhibitor mixture (Beyotime Biotechnology, Shanghai, China). The Flag‐beads were added to the supernatant of total protein and incubated together overnight at 4°C, and the elution protein was obtained by the same method as Co‐IP. The signals of phosphorylation were detected with antibody anti‐pSer/pThr (PP2551; ECM Biosciences, Versailles, KY, USA).

### Analysis of sugars via gas chromatography method

The gas chromatography (GC) assay was conducted according to a previously published method (Li et al., 2020a; Mao et al., 2024). Sugars of plants were extracted in 75% (v/v) methanol, with ribitol added as internal standard. Each sample was dried under vacuum without heat, and then derivatized with methoxyamine hydrochloride and *N*‐methyl‐*N*‐trimethylsilyl‐trifluoroacetamide sequentially. The sugar content was analyzed using the The GC 9720plus Gas Chromatography Workstation (FULI Instruments, Zhejiang, China) with a flame ionization detector and a non‐polar RBX‐5 (5%‐phenyl)‐methylpolysiloxane column (30.0 m × 0.32 mm × 0. 5 μm).

### CsTST2 transport activity assay in *X. laevis* oocyte cells

The sequences of *CsTST2*, *CsTST2* ^*3M*^, and *CsCIPK23* were amplified and cloned into *X. laevis* oocyte expression vectors (with or without N‐terminal YFP) by an advanced uracil excision‐based cloning technique previously described (Nour‐Eldin et al., 2006; Li et al., 2020b). The cRNA was synthesized using the mMessage mMachine kit (T7; Thermo Fisher, Waltham, MA, USA) and 50 nL of cRNA was injected into each oocyte cell for 2 d of protein expression. The YFP signal in the oocyte was detected using a confocal microscope (TCS SP8; Leica, Mannheim, Germany) with PM stain (Deep Red; Thermo Fisher, Waltham, MA, USA) as a marker for the colocalization of the protein in the membrane. YFP and Deep Red were excited at 514 and 649 nm, and the emission signals were detected at 520–540 and 650–700 nm, respectively. Two‐electrode voltage‐clamp recordings were conducted in oocyte cells that were either nonloaded or loaded with 50 nL of 100 mmol/L sucrose or glucose 2 h prior to the recordings under constant perfusion in ND96 solution consisting of 96 mmol/L NaCl, 1 mmol/L KCl, 1.8 mmol/L CaCl_2_, pH 5.5 or pH 7.5. Currents were elicited by voltage pulses stepped between +40 and −180 mV in 20‐mV increments. The AxoClamp 900 A amplifier and pCLAMP software (version 11.2; Axon Instruments, Molecular Devices, San Jose, CA, USA) were used to acquire and analyze the whole‐cell currents. Based on a previous report (Li et al., 2023b), the oocyte with the expression of CsTST2 and nuclease‐free water‐injected control cells were used for sugar efflux assay. Each oocyte cell was injected with 100 nL 100 mmol/L ^13^C‐glucose or ^13^C‐sucrose and immediately transferred into ND96 buffer (pH 5.5). After incubating at 18°C for 4 h, the oocytes were washed three times with ND96 solution buffer. Then four oocyte cells were set one each sample, which was transferred to a pressed tin foil cup for oven drying. The ^13^C labeled sugars level was measured by using isotope ratio MS (Isoprime 100; Elementar, Germany). Primers used in this study are listed in Table S2.

### Statistical analysis

All experiments in this study were repeated at least three times. Analysis of variance followed by Tukey's Honestly Significant Difference test was conducted using Sigmaplot 11.0 (Systat Software).

### Accession Numbers

Gene IDs from this study can be found in CPBD (http://citrus.hzau.edu.cn/): *CsTST1* (Cs3g27610), *CsTST2* (Cs8g01220), *CsCBL1* (Cs5g07690), *CsCIPK23* (Cs2g08190).

## CONFLICTS OF INTEREST

The authors declare no conflict of interest.

## AUTHOR CONTRIBUTIONS

C.L. and J.H.L. conceived and designed the research. M.L. performed most of the experiments and analyzed the data, Z.M., Z.Z., S.G., Y.L., Z.L., X.S., and X.Z. assisted the experiments. M.L. and C.L. wrote the manuscript draft, C.L. and J.H.L. finalized the writing and revision of the manuscript. All authors read and approved of its content.

## Supporting information

Additional Supporting Information may be found online in the supporting information tab for this article: http://onlinelibrary.wiley.com/doi/10.1111/jipb.13812/suppinfo

**Figure S1.** Phylogenetic analysis of sweet orange (*Citrus sinensis*) tonoplast sugar transporter (TST) members and *CsTST1* expression assay
**Figure S2.** The sub‐cell localization of *Citrus sinensis* tonoplast sugar transporter 2–green fluorescent protein (CsTST2‐GFP) protein in yeast cells
**Figure S3.** The expression and localization of *Citrus sinensis* tonoplast sugar transporter 2–yellow fluorescent protein (CsTST2‐YFP) fusion protein in oocytes
**Figure S4.** ^13^C‐glucose and ^13^C‐sucrose efflux activity of *Citrus sinensis* tonoplast sugar transporter 2 (CsTST2) in *Xenopus* oocyte
**Figure S5.** The transient overexpression of *Citrus sinensis tonoplast sugar transporter 2* (*CsTST2*) induces sugar accumulation in the juice sac
**Figure S6.** The sucrose content in wild type (WT) and *Citrus sinensis tonoplast sugar transporter 2* – overexpression (*CsTST2*‐OE) tomato fruits
**Figure S7.** The identified calcineurin B‐like protein kinase (CIPK) members in sweet orange (*Citrus sinensis*)
**Figure S8.** The DUAL membrane system of yeast two‐hybrid (Y2H) assay between *Citrus sinensis tonoplast sugar transporter 2* (CsTST2) and *C. sinensis* calcineurin B‐like protein kinases (CsCIPKs)
**Figure S9.** The expression heat map of *Citrus sinensis calcineurin B‐like protein kinase* (*CsCIPK*) members based on the previous RNA sequencing data in citrus juice sacs
**Figure S10.** The expression of *Citrus sinensis calcineurin B‐like protein kinase 23* (*CsCIPK23*) during the sweet orange fruit development
**Figure S11.** Schematic diagram showing the transmembrane and loop regions of *Citrus sinensis* tonoplast sugar transporter 2 (CsTST2) protein
**Figure S12.** The identified calcineurin B‐like protein (CBL) members in sweet orange (*Citrus sinensis*)
**Figure S13.** The expression heat map of *Citrus sinensis calcineurin B‐like* (*CsCBL*) members based on the previous RNA sequencing data in citrus juice sacs
**Figure S14.** *Citrus sinensis* calcineurin B‐like (CBL) protein kinase 23 (CsCIPK23) interacts with CsCBL1
**Figure S15.** The subcellular localization of *Citrus sinensis* calcineurin B‐like 1 (CsCBL1) protein
**Figure S16.** The phosphorylation sites of *Citrus sinensis* tonoplast sugar transporter 2 (CsTST2) protein by liquid chromatography with tandem mass spectrometry (LC‐MS/MS) analysis
**Figure S17.** The *Citrus sinensis* tonoplast sugar transporter 2 (CsTST2^3M^) protein was localized in the vacuole membrane
**Figure S18.** The expression level of *Citrus sinensis tonoplast sugar transporter 2* (*CsTST2*) in *CsTST2*‐OE (overexpression) and *CsTST2* ^*3M*^‐OE calli
**Figure S19.** The expression of *Citrus sinensis tonoplast sugar transporter 2* (*CsTST2*) and *Citrus sinensis calcineurin B‐like protein kinase 23* (*CsCIPK23*) in *CsCIPK23*‐OE (overexpression)/*CsTST2*‐RNA interference calli
**Figure S20.** The comparison of tonoplast sugar transporter (TST) proteins' phosphorylation sites in different species

**Table S1.** List of genes were identified by DUAL membrane yeast two‐hybrid (Y2H) system
**Table S2.** List of primers used in this study
