# Supplementary material for: CBL1/CIPK23 phosphorylates tonoplast sugar transporter TST2 to enhance sugar accumulation in sweet orange (Citrus sinensis)
Source: J Integr Plant Biol. 2024 Nov 29;67(2):327–44. doi: 10.1111/jipb.13812 (PMC11814916; doi:10.1111/jipb.13812)
Supplement: Supplementary file 1 — Figure S1. Phylogenetic analysis of sweet orange (Citrus sinensis) tonoplast sugar transporter (TST) members and CsTST1 expression assay Figure S2. The sub‐cell localization of Citrus sinensis tonoplast sugar transporter 2–green fluorescent protein (CsTST2‐GFP) protein in yeast cells Figure S3. The expression and localization of Citrus sinensis tonoplast sugar transporter 2–yellow fluorescent protein (CsTST2‐YFP) fusion protein in oocytes Figure S4. 13C‐glucose and 13C‐sucrose efflux activity of Citrus sinensis tonoplast sugar transporter 2 (CsTST2) in Xenopus oocyte Figure S5. The transient overexpression of Citrus sinensis tonoplast sugar transporter 2 (CsTST2) induces sugar accumulation in the juice sac Figure S6. The sucrose content in wild type (WT) and Citrus sinensis tonoplast sugar transporter 2 – overexpression (CsTST2‐OE) tomato fruits Figure S7. The identified calcineurin B‐like protein kinase (CIPK) members in sweet orange (Citrus sinensis) Figure S8. The DUAL membrane system of yeast two‐hybrid (Y2H) assay between Citrus sinensis tonoplast sugar transporter 2 (CsTST2) and C. sinensis calcineurin B‐like protein kinases (CsCIPKs) Figure S9. The expression heat map of Citrus sinensis calcineurin B‐like protein kinase (CsCIPK) members based on the previous RNA sequencing data in citrus juice sacs Figure S10. The expression of Citrus sinensis calcineurin B‐like protein kinase 23 (CsCIPK23) during the sweet orange fruit development Figure S11. Schematic diagram showing the transmembrane and loop regions of Citrus sinensis tonoplast sugar transporter 2 (CsTST2) protein Figure S12. The identified calcineurin B‐like protein (CBL) members in sweet orange (Citrus sinensis) Figure S13. The expression heat map of Citrus sinensis calcineurin B‐like (CsCBL) members based on the previous RNA sequencing data in citrus juice sacs Figure S14. Citrus sinensis calcineurin B‐like (CBL) protein kinase 23 (CsCIPK23) interacts with CsCBL1 Figure S15. The subcellular localizatio [file JIPB-67-327-s002.docx]

**Figure S1. Phylogenetic analysis of sweet orange (C. sinensis) TST members and CsTST1 expression assay. (A)** Phylogenetic analysis of CsTSTs (starred) and other homologous proteins using the MEGA software. **(B)** The expression of *CsTST1* during the citrus fruit development. Values represents the mean ± SE with three biological replicates.

**Figure S2. The sub-cell localization of CsTST2-GFP protein in yeast cell.** The signal was observed by confocal microscopy. Scale bars = 5 μM.


**Figure S3. The expression and localization of CsTST2-YFP fusion protein in oocyte.** Scale bars = 200 μm.

**Figure S4. 13C-glucose and 13C-sucrose efflux activity of CsTST2 in *Xenopus* oocytes.** Oocytes were injected with 100 nl of 100 mM 13C-glucose or 13C-sucrose and incubated in ND96 buffer at pH 5.5 for 4 hours. The sample consisted of four oocytes. The value was isotope relative ratio (δ ‰) and represents the mean ± SE with three biological replicates. Statistics analysis, the asterisks indicate significant differences as assessed by independent samples t-test, ****P* < 0.001.

**Figure S5. The transient overexpression of *CsTST2* induces the sugar accumulation in juice sac. (A)** The relative expression of *CsTST2* in juice sac samples. **(B-D)** The fructose **(B)**, glucose **(C)** and sucrose **(D)** content in juice sac samples. The empty vecter of overexpression is pK7WG2D (EV). Value represents the mean ± SE with three biological replicates. Statistics analysis by independent samples t-test (**P* < 0.05, ***P* < 0.01). FW in (B-D): Fresh Weight

**Figure S6. The sucrose content in WT and *CsTST2*-OE tomato fruits. (A)** The sucrose content of *CsTST2-OE* tomato fruits was no-significant in comparison with EV control. Value represents the mean ± SE with three biological replicates. Statistics analysis by one-way ANOVA with Tukey’s test. **(B)** The fruit photos of EV control and *CsTST2*-OE lines. Scale bars = 2 cm. FW in (A): Fresh Weight.

**Figure S7. The identified CIPK members in sweet orange (*C. sinensis*).** Phylogenetic analysis of CIPK members in sweet orange (*C. sinensis*) and *Arabidopsis* based on amino acid sequence alignment by the Maximum Likelihood method using MEGA. Scale length = 0.2. Sixteen CIPKs of sweet orange (*C. sinensis*) were named for their homology with *Arabidopsis* CIPK members.

**Figure S8. The DUAL membrane system of Y2H assay between CsTST2 and CsCIPKs.** The sixteen CDS of *CsCIPKs* were cloned into pPR3-N vector, and each then co-transformed into yeast NMY51 cells with *CsTST2*-pBT3-STE. Plasmids containing the pNubG-*Fe65*/pTSU2-*APP* and *CsTST2*-pBT3-STE/pPR3-N were used as positive and negative control, respectively. Growth plaques in SD/-Leu-Trp-Ura-His-Ala medium indicate an interaction between two proteins. Droplet test with 0.2 (OD_600_) of yeast cells.

**Figure S9. The expression heat map of *CsCIPK* members based on the previous RNA-seq data in citrus juice sacs.** The value was log of FPKM. The raw RNA-seq data have been uploaded to the Gene Expression Omnibus (GEO) database of the NCBI (accession number: GSE125726) (Feng et al., 2022).

**Figure S10. The expression of *CsCIPK23* during the sweet orange fruit development.** Values represent the mean ± SE with three biological replicates. DAF: Days After Flowering.

**Figure S11. Schematic diagram showing the transmembrane and loop regions of CsTST2 protein.** Blue column and blue lines indicate the CsTST2 transmembrane part (TM1 –TM12) and cytosolic region (loop) respectively.

**Figure S12. The identified CBL members in sweet orange (*C. sinensis*).** Phylogenetic analysis of CBL members in sweet orange (*C. sinensis*) and *Arabidopsis* based on amino acid sequence alignment by the Maximum Likelihood method using MEGA64. Sixteen CsCBL members were named for their homology with *Arabidopsis* CBLs.

**Figure S13. The expression heat map of *CsCBL* members based on the previous RNA-seq data in citrus juice sacs.** The value was log of FPKM. The raw RNA-seq data have been uploaded to the Gene Expression Omnibus (GEO) database of the NCBI (accession number: GSE125726) (Feng et al., 2022).

**Figure S14. CsCIPK23 interacts with CsCBL1. (A)** The Y2H assay between CsCIPK23 and CsCBLs. The sixteen CDS of *CsCBLs* were cloned into pGADT7 vector, and each then co-transformed into yeast Y2H-gold cells with *CsCIPK23*-pGBKT7. The yeast with the expression of pGBKT7-*53*/pGADT7-*T* and *CsCIPK23*-pGBKT7/pGADT7 were used as positive and negative control, respectively. Growth plaques in SD/-His-Leu-Trp-Ura-Ala medium indicate an interaction between two proteins. Droplet test with 0.2 (OD600) of yeast cells. **(B)** The LCI assay between CsCIPK23 and CsCBLs in *N. benthamiana* leaves. CsCIPK23 interacts with CsCBL1 but not interact with CsCBL2/3/7/9/15. The leaves were containing different pairs of constructs as indicated, and no fluorescence signals.

**Figure S15. The subcellular localization of CsCBL1 protein. (A)** Images of confocal microscopy were epidermal cells expressing the empty vector control (cytosolic) and CsCBL1 tagged with GFP fluorescent proteins in *N. benthamiana*, respectively. **(B)** An isolated vacuole of tobacco expressing CsCBL1-GFP fluorescent proteins. Scale bars = 10 μm. AtCBL1 was used as plasma membrane (PM) marker.

**S-Figure 16. The phosphorylation sites of CsTST2 protein by LC–MS/MS analysis. (A-D)** The Ser277 **(A)**, Ser337 **(B)**, Ser354 **(C)** and Ser440 **(D)** residues of CsTST2 were identified to be phosphorylated by CsCIPK23. The phosphorylated Ser residues in the CsTST2 fragment are highlighted in red.

**Figure S17. The CsTST2^3M^ protein was localized in the vacuole membrane.** Scale bars = 10 μm.

**Figure S18. The expression level of *CsTST2* in *CsTST2*-OE and *CsTST2^3M^*-OE calli.** The bars represent the mean value ± SE with three biological replicates, Statistical significance was determined using one-way ANOVA with Tukey’s test (**P* < 0.05, ***P* < 0.01, ****P* < 0.001).

**Figure S19. The expression of *CsTST2* and *CsCIPK23* in *CsCIPK23*-OE/*CsTST2*-RNAi calli. (A-B)** The expression of *CsTST2* **(A)** and *CsCIPK23* **(B)** by RT-qPCR analysis in citrus calli lines, which were transiently overexpress *CsCIPK23* in transgenic tissue-cultured citrus lines of empty vector control and *CsTST2*-silenced background. Bars represent the mean value ± SE with three biological replicates. Different letters indicate significant differences as assessed by one-way ANOVA (with Tukey’s test), *P* < 0.05.

**Figure S20. The comparison of TST proteins’ phosphorylation sites in different species.** Protein sequence alignment of CsTST2 and homologs in the indicated species via MEGA and GENEDOC.
