# Supplementary material for: CBL1/CIPK23 phosphorylates tonoplast sugar transporter TST2 to enhance sugar accumulation in sweet orange (Citrus sinensis)
Source: J Integr Plant Biol. 2024 Nov 29;67(2):327–44. doi: 10.1111/jipb.13812 (PMC11814916; doi:10.1111/jipb.13812)
Supplement: Supplementary file 2 — Table S1. List of genes were identified by DUAL membrane yeast two‐hybrid (Y2H) system Table S2. List of primers used in this study [file JIPB-67-327-s001.pdf]

Table S1. List of gene were identified by DUAL membrane Y2H system		
Gene ID	Length (bp)	Name
Cs6g04650	675	lipid phosphate phosphatase gamma
Cs2g01050	519	zinc finger A20 and AN1 domain-containing stress-associated protein 8
Cs2g08190	1395	CBL-INTERACTING PROTEIN KINASE 23
Cs7g04580	678	Glutathione S-transferase U17
Cs7g10860	747	Interacts with TATA-box binding protein 2
Cs1g12560	708	26.5 kDa heat shock protein
Cs3g18310	2619	Synaptonemal complex protein 1
Cs3g12980	1269	IQ calmodulin-binding motif family protein
Cs8g10260	1473	DNAJ heat shock N-terminal domain-containing protein
Cs7g07780	597	No Hit Found
Cs3g13440	1626	Heavy metal transport/detoxification domain-containing protein
Cs3g21500	540	Abscisic stress ripening-like protein
Cs8g16300	2184	E3 ubiquitin-protein ligase WAV3
Cs5g31990	594	Methyl-CpG-binding domain-containing protein 4

Table S2. List of primers used in this study.

	Primer name	Primers sequence(5'-3')
**Gene expression and cloning**		
RT-qPCR	*q-Actin* -F	CCGACCGTATGAGCAAGGAAA
	q-Actin -R	TTCCTGTGGACAATGGATGGA
	q-CsTST1 -F	CTGTGCCATGGCTTACTGGA
	q-CsTST1 -R	GCATAAACACCGAACGCACC
	q-CsTST2 -F	TGCTTTGTCATGGGTTTTGGG
	q-CsTST2 -R	CAATGCAGATGCCACGTACC
	q-CIPK23 -F	TGTGACACGAATAACGATGGC
	q-CIPK23 -R	TCCACAACAAGGTTTCGAGAAT
Subcellular localization	*CsTST2* -GFP101-F	atgggatctactagtgaattcATGGGGGGAGCTGCGCTT
	CsTST2 -GFP101-R	ggggtaccgtcgacggatccGTTGTTCTTGGCAGCATCAGC
Overexpression	*CsTST2* -pE-Sal1-F	aaaggaaccaattcagtcgacATGGGGGGAGCTGCGCTT
	CsTST2 -pE-Kpn1-R	tggaaaagggaattcggtaccGTTGTTCTTGGCAGCATCAGC
	CsCIPK23 -PE-Sal1-F	aaaggaaccaattcagtcgacATGGCTTCTCGCACAGGCG
	CsCIPK23 -PE-Kpn1-R	tggaaaagggaattcggtaccGTGGCTAACAAATAAATATGCAGC
	CsCIPK23 -GW-F	GGGGACAAGTTTGTACAAAAAAGCAGGCTTAATGGCTTCTCGCACAGGCG
	CsCIPK23 -GW-R	GGGGACCACTTTGTACAAGAAAGCTGGGTTGTGGCTAACAAATAAATATGCAGC
RNAi	*CsTST2* -GW-F	GGGGACAAGTTTGTACAAAAAAGCAGGCTTCATGGGGGGAGCTGCGCTT
	CsTST2 -GWRi-R-NO	GGGGACCACTTTGTACAAGAAAGCTGGGTCAGATGAGATTATTAACATTGGACGGC
	CsCIPK23 -Ri-F	GGGGACAAGTTTGTACAAAAAAGCAGGCTTCATGGCTTCTCGCACAGGCG
	CsCIPK23 -Ri-R	GGGGACCACTTTGTACAAGAAAGCTGGGTCTTGCCATCACCTCATACATGCG
Blunt-clone vector	*CsCIPK23* -B-KpnI-F	GGTACCATGGCTTCTCGCACAGGCG
	CsCIPK23 -B-SalI-R	GTCGACCTAGTGGCTAACAAATAAATATGCAGCACC
Oocyte expression	*CsTST2* -U-F	GGCTTAA/ideoxyU/ATGGGGGGAGCTGCGCTTGTTGC
	CsTST2 -U-R	GGTTTAA/ideoxyU/TCAGTTGTTCTTGGCAGCATCAG
	CsCIPK23 -U-F	GGCTTAA/ideoxyU/ATGGCTTCTCGCACAGGCGGAGC
	CsCIPK23 -U-R	GGTTTAA/ideoxyU/CTAGTGGCTAACAAATAAATATG
	CsTST2 -U-R-NO	GGTTTAA/ideoxyU/GTTGTTCTTGGCAGCATCAGCCT
**Yeast assay**		
Overexpression	*CsTST2* -p196-EcoR1-F	tcccccgggctgcaggaattcATGGGGGGAGCTGCGCTT
	CsTST2 -p196-Sal1-R	gggcccccctcgaggtcgacTCAGTTGTTCTTGGCAGCATCAGC
Subcellular localization	*CsTST2* -PYGFP-F	ctcgattctacgcgtaccggtATGGGGGGAGCTGCGCTT
	CsTST2 -PYGFP-R	gtgatggtgatgatgaccggtTCAGTTGTTCTTGGCAGCATCA
DUAL membrane system	*CsTST2* -pBT3S-F	attaacaaggccattacggccGGGGGAGCTGCGCTT
	CsTST2 -pBT3S-R	aactgattggccgaggcggccccGTTGTTCTTGGCAGCATCA
	CsCIPK10-pPR-F	gtatcaacgcagagtggccattacggccATGTTTGGGATGGATCATAATAAGC
	CsCIPK10 -pPR-R	atcgaattctcgagaggccgaggcggccTTATTGCTGCTGCTGCTGTTCT
	CsCIPK15 -pPR-F	gtatcaacgcagagtggccattacggccATGGAGAACAAAGGGAGTATATTGATG
	CsCIPK15 -pPR-R	atcgaattctcgagaggccgaggcggccCTATGAGTCTTGAGGAGGGGCA
	CsCIPK18 -pPR-F	gtatcaacgcagagtggccattacggccATGGCGCACGCCACCGCG
	CsCIPK18 -pPR-R	atcgaattctcgagaggccgaggcggccCTATTCAGTGTCTGATGGCAAATGC
	CsCIPK20 -pPR-F	gtatcaacgcagagtggccattacggccATGGAGAAGAAAGCGGGAATTT
	CsCIPK20 -pPR-R	atcgaattctcgagaggccgaggcggccTTACTGCTCATCATTTCCTTGCC
	CsCIPK23 -pPR-F	gtatcaacgcagagtggccattacggccATGGCTTCTCGCACAGGCG
	CsCIPK23 -pPR-R	atcgaattctcgagaggccgaggcggccCTAGTGGCTAACAAATAAATATGCAGC
	CsCIPK11 -pPR-F	gtatcaacgcagagtggccattacggccATGTCAGAGATCGAACGACACAAT
	CsCIPK11 -pPR-R	atcgaattctcgagaggccgaggcggccTCAGTTACCGGAAACCGTATCTC
	CsCIPK3 -pPR-F	gtatcaacgcagagtggccattacggccATGAGTCAACCTAAAATTAAACGCC
	CsCIPK3 -pPR-R	atcgaattctcgagaggccgaggcggccTTATTTCATTTCCTGCATATCCTCC
	CsCIPK6 -pPR-F	gtatcaacgcagagtggccattacggccATGGCTACATCAAACAGTAACGGC
	CsCIPK6 -pPR-R	atcgaattctcgagaggccgaggcggccTTAAATTTGTTGCTCCTCCAGGT
	CsCIPK24 -pPR-F	gtatcaacgcagagtggccattacggccATGAAGAAGCAAACTAGGAGGGTT
	CsCIPK24 -pPR-R	atcgaattctcgagaggccgaggcggccTCAGCAACTCATTGTCCTAAGCA
	CsCIPK14 -pPR-F	gtatcaacgcagagtggccattacggccATGGGTGATAACACTGGCGATAT
	CsCIPK14 -pPR-R	atcgaattctcgagaggccgaggcggccTCACTGCGAGACCCCGGC
	CsCIPK16 -pPR-F	gtatcaacgcagagtggccattacggccATGGAAGAGGAGAGGCATGTTCT
	CsCIPK16 -pPR-R	atcgaattctcgagaggccgaggcggccTTAATTTAGTTGGTAGGCAGCATTTG
	CsCIPK2 -pPR-F	gtatcaacgcagagtggccattacggccATGGAGACTAAGGGGAAGGTGTT
	CsCIPK2 -pPR-R	atcgaattctcgagaggccgaggcggccTTATATGGAATGCACTTGCTGCA
	CsCIPK1 -pPR-F	gtatcaacgcagagtggccattacggccATGGTGATAGCAGAGAAGAAGCAGG
	CsCIPK1 -pPR-R	atcgaattctcgagaggccgaggcggccTCACACTTCTGTGGCTAGCAACTC
	CsCIPK4 -pPR-F	gtatcaacgcagagtggccattacggccATGGAACCAGGCTGTTCTCCA
	CsCIPK4 -pPR-R	atcgaattctcgagaggccgaggcggccTCACATCACATCACTATTATGCCAA
	CsCIPK5 -pPR-F	gtatcaacgcagagtggccattacggccATGAAATTTGAGACTATGGATGAGCG
	CsCIPK5 -pPR-R	atcgaattctcgagaggccgaggcggccCTATTGAGAGTTGTTTTCACCTTGCC
	CsCIPK9 -pPR-F	gtatcaacgcagagtggccattacggccATGAGCGTGAAAGTACCGGC
	CsCIPK9 -pPR-R	atcgaattctcgagaggccgaggcggccTCACGCCCTTAATTCTTCTTTATTT
Y2H	*CsCIPK23* -pGBKT7-F	atggccatggaggccgaattcATGGCTTCTCGCACAGGCG
	CsCIPK23 -pGBKT7-R	ccgctgcaggtcgacggatccGTGGCTAACAAATAAATATGCAGC
	CsCBL1 -pGADT7-F	gccatggaggccagtgaattcATGGGCTGTTTTCAGTCTAAAGTAGC
	CsCBL1 -pGADT7-R	cagctcgagctcgatggatccTTATGTAGCAATCTCATCGACTTCTGAG
	CsCBL2 -pGADT7-F	gccatggaggccagtgaattcATGGTGCAGTGCCTAGACGG
	CsCBL2 -pGADT7-R	cagctcgagctcgatggatccTCAGGTATCGTCAACTTGCGAG
	CsCBL3 -pGADT7-F	gccatggaggccagtgaattcATGGATTCTGCAATGAACTCTTTCA
	CsCBL3 -pGADT7-R	cagctcgagctcgatggatccTTACATCTCAAATTCTGTGTCATCCA
	CsCBL4 -pGADT7-F	gccatggaggccagtgaattcATGGGTTGCGTCTTGACAAAG
	CsCBL4 -pGADT7-R	cagctcgagctcgatggatccCTACATTTCTGAGTCTTCAACTTCAGAA
	CsCBL5 -pGADT7-F	gccatggaggccagtgaattcATGGGCTGTGTTTGCATGAAG
	CsCBL5 -pGADT7-R	cagctcgagctcgatggatccTTACAGGAAGCTGTTAATGTCATCTTC
	CsCBL6 -pGADT7-F	gccatggaggccagtgaattcATGTCGAAAGTGAGTTTCCTCAATT
	CsCBL6 -pGADT7-R	cagctcgagctcgatggatccTTAAGCCACTTTCACATTGCGA
	CsCBL7 -pGADT7-F	gccatggaggccagtgaattcATGTTGCAGTGCATAGAGGGATT
	CsCBL7 -pGADT7-R	cagctcgagctcgatggatccTTAGGTATCTTCAACTCTTGAATGGAA
	CsCBL8 -pGADT7-F	gccatggaggccagtgaattcATGAATGCTTTTGGGAGATGCT
	CsCBL8 -pGADT7-R	cagctcgagctcgatggatccCTAATATCGAGCTTCACTGTGCAGC
	CsCBL9 -pGADT7-F	gccatggaggccagtgaattcATGTGCCCGACCGGTACC
	CsCBL9 -pGADT7-R	cagctcgagctcgatggatccTCAGGCAACAGTATCAACGGC
	CsCBL10 -pGADT7-F	gccatggaggccagtgaattcATGGATTCTTCCGCCAACCG
	CsCBL10 -pGADT7-R	cagctcgagctcgatggatccTCAGTCTTCAACCTCGGTGTTAAA
	CsCBL11 -pGADT7-F	gccatggaggccagtgaattcATGGGAGGTATAGTAGGGAAGCC
	CsCBL11 -pGADT7-R	cagctcgagctcgatggatccTTAATTCTTCTCTTCCCCTTCTTCC
	CsCBL12 -pGADT7-F	gccatggaggccagtgaattcATGAGATGGAAGCTTACTGGACATT
	CsCBL12 -pGADT7-R	cagctcgagctcgatggatccTCACTGAACATGAACTTTTGAATTCA
	CsCBL13 -pGADT7-F	gccatggaggccagtgaattcATGGCTCCTCTTACCGCCA
	CsCBL13 -pGADT7-R	cagctcgagctcgatggatccTTAAGAAACAGTACTACGAAGCATCATG
	CsCBL14 -pGADT7-F	gccatggaggccagtgaattcATGGGCAACGCGTCGTCG
	CsCBL14 -pGADT7-R	cagctcgagctcgatggatccCTAGTCCACTGGAACCTCTACTTCG
	CsCBL15 -pGADT7-F	gccatggaggccagtgaattcATGAAACTCATCGAGAAGCTCAGC
	CsCBL15 -pGADT7-R	cagctcgagctcgatggatccTCATCTCTGCAACTCCATCATCC
	CsCBL16 -pGADT7-F	gccatggaggccagtgaattcATGGGGACTGCCTCGTCTATG
	CsCBL16 -pGADT7-R	cagctcgagctcgatggatccTTAGTCAACTGGAACTTCAACCTCC
**BiFC assay**		
	CsTST2 -BiFC-BamH1-F	tggcgcgccactagtggatccATGGGGGGAGCTGCGCTT
	CsTST2 -BiFC-Sal1-R	agcggtaccctcgaggtcgacGTTGTTCTTGGCAGCATCAGC
	CsCIPK23 -BiFC-BamH1-F	tggcgcgccactagtggatccATGGCTTCTCGCACAGGCG
	CsCIPK23 -BiFC-Sal1-R	agcggtaccctcgaggtcgacGTGGCTAACAAATAAATATGCAGCA
	CsCIPK9 -BiFC--BamH1-F	tggcgcgccactagtggatccATGAGCGTGAAAGTACCGGC
	CsCIPK9 -BiFC--Sal1-R	agcggtaccctcgaggtcgacCGCCCTTAATTCTTCTTTATTT
**LCI assay**		
	CsCIPK23 -772-KpnI-F	TACGCGTCCCGGGGCGGTACCATGGCTTCTCGCACAGGCG
	CsCIPK23 -772-SalI-R	ACGAAAGCTCTGCAGGTCGACCTAGTGGCTAACAAATAAATATGCAGC
	CsTST2 -771- KpnI-F	ACGGGGGACGAGCTCGGTACCATGGGGGGAGCTGCGCTT
	CsTST2 -771-SalI-R	CGCGTACGAGATCTGGTCGACGTTGTTCTTGGCAGCATCA
	CsCBL1 -771-KpnI-F	ACGGGGGACGAGCTCGGTACCATGGGCTGTTTTCAGTCTAAAGTAGC
	CsCBL1 -771-SalI-R	CGCGTACGAGATCTGGTCGACTGTAGCAATCTCATCGACTTCTGAG
	CsCBL2 -771-KpnI-F	ACGGGGGACGAGCTCGGTACCATGGTGCAGTGCCTAGACGG
	CsCBL2 -771-SalI-R	CGCGTACGAGATCTGGTCGACGGTATCGTCAACTTGCGAG
	CsCBL3 -771-KpnI-F	ACGGGGGACGAGCTCGGTACCATGGATTCTGCAATGAACTCTTTCA
	CsCBL3 -771-SalI-R	CGCGTACGAGATCTGGTCGACCATCTCAAATTCTGTGTCATCCA
	CsCBL7 -771-KpnI-F	ACGGGGGACGAGCTCGGTACCATGTTGCAGTGCATAGAGGGATT
	CsCBL7 -771-SalI-R	CGCGTACGAGATCTGGTCGACGGTATCTTCAACTCTTGAATGGAACA
	CsCBL9 -771-KpnI-F	ACGGGGGACGAGCTCGGTACCATGTGCCCGACCGGTACC
	CsCBL9 -771-SalI-R	CGCGTACGAGATCTGGTCGACGGCAACAGTATCAACGGC
	CsCBL15 -771-KpnI-F	ACGGGGGACGAGCTCGGTACCATGAAACTCATCGAGAAGCTCAGC
	CsCBL15 -771-SalI-R	CGCGTACGAGATCTGGTCGACTCTCTGCAACTCCATCATCCTTG
	CsCIPK9 -772-KpnI-F	TACGCGTCCCGGGGCGGTACCATGAGCGTGAAAGTACCGGC
	CsCIPK9 -772-SalI-R	ACGAAAGCTCTGCAGGTCGACTCACGCCCTTAATTCTTCTTTATTT
**Co-IP assay**		
	CsTST2^{loop} - GW-*F*	GGGGACAAGTTTGTACAAAAAAGCAGGCTTCATGTGGCTTGTGAGTAAAGGGC
	CsTST2^{loop} - GW-*R*	GGGGACCACTTTGTACAAGAAAGCTGGGTCAAGTATTTGAATGCCCACACCC
**Pulldown assay**		
	CsCIPK23 -4T-EcoR1-F	ccgcgtggatccccggaattcATGGCTTCTCGCACAGGCG
	CsCIPK23 -4T-Sal1-R	ggtttgcacgctcgagtcgacGTGGCTAACAAATAAATATGCAGCA
	CsTST2^{loop} -32a-BamH1-F	gccatggctgatatcggatccATGTGGCTTGTGAGTAAAGGGC
	CsTST2^{loop} -32a-Sal1-R	tgcggccgcaagcttgtcgacAAGTATTTGAATGCCCACACCC
	CsCBL1 -32a-BamH1-F	gccatggctgatatcggatccATGGGCTGTTTTCAGTCTAAAGTAGC
	CsCBL1 -32a-Sal1-R	tgcggccgcaagcttgtcgacTGTAGCAATCTCATCGACTTCTGAG
**Overlap PCR for *CsTST2* site mutation**		
	277A-R1	AATTGCCTGTCCAGTGACTGGTCTAGCAATC
	277A-F2	CAGTCACTGGACAGGCAATTGTTGGTCTAGGGTCTAGGCA
	337A-R1	ACAAGTGCCTCCTCATCCCACTCTTCATTT
	337A-F2	TGGGATGAGGAGGCACTTGTCAGAGAGGGTGACGAATATC
	354A-R1	CATCTGCGTCACCACCAGCAGCATCAGATG
	354A-F2	TGCTGGTGGTGACGCAGATGACAATTTGCAGAGCCCATTG
	440A-R1	GATGTGCTGCAGGGACACCCTCCTGGTGCA
	440A-F2	GGGTGTCCCTGCAGCACATCGTGGATCCTTAGTTTCTATG
